# Thorax-Segment- and Leg-Segment-Specific Motor Control for Adaptive Behavior

**DOI:** 10.3389/fphys.2022.883858

**Published:** 2022-05-04

**Authors:** Elzbieta Hammel, Charalampos Mantziaris, Joscha Schmitz, Ansgar Büschges, Matthias Gruhn

**Affiliations:** Animal Physiology, Biocenter, Mathematisch-Naturwissenschaftliche Fakultät, Universität zu Köln, Cologne, Germany

**Keywords:** motor control, turning, modulation, insect, locomotion, walking, adaptive behavior

## Abstract

We have just started to understand the mechanisms underlying flexibility of motor programs among segmental neural networks that control each individual leg during walking in vertebrates and invertebrates. Here, we investigated the mechanisms underlying curve walking in the stick insect *Carausius morosus* during optomotor-induced turning. We wanted to know, whether the previously reported body-side specific changes in a two-front leg turning animal are also observed in the other thoracic leg segments. The motor activity of the three major leg joints showed three types of responses: 1) a context-dependent increase or decrease in motor neuron (MN) activity of the antagonistic MN pools of the thorax-coxa (ThC)-joint during inside and outside turns; 2) an activation of 1 MN pool with simultaneous cessation of the other, independent of the turning direction in the coxa-trochanteral (CTr)-joint; 3) a modification in the activity of both FTi-joint MN pools which depended on the turning direction in one, but not in the other thorax segment. By pharmacological activation of the meso- or metathoracic central pattern generating networks (CPG), we show that turning-related modifications in motor output involve changes to local CPG activity. The rhythmic activity in the MN pools of the ThC and CTr-joints was modified similarly to what was observed under control conditions in saline. Our results indicate that changes in meso- and metathoracic motor activity during curve walking are leg-joint- and thorax-segment-specific, can depend on the turning direction, and are mediated through changes in local CPG activity.

## Introduction

Rhythmic locomotor activity in both vertebrates and invertebrates results from interactions between the activity of central pattern generating networks (CPGs) and sensory feedback that tunes the activity to the demands of the environment (Grillner, 2003; Büschges and Gruhn, 2008; Bidaye et al., 2018; Akay, 2020; Akay and Murray, 2021). Particularly insects have been used for long to investigate the neuronal control of coordinated limb movements. This has led to a good understanding of the basic principles underlying simple stepping patterns and straight walking. We know, for example, that the activity of motor neurons during leg stepping is generated by tonic excitation, shaped by inhibition related to CPG activity. Its magnitude and phase are modulated by position and load feedback from the own and from neighboring legs (e.g., Büschges et al., 2004; Ludwar et al., 2005a; Ludwar et al., 2005b; Büschges et al., 2008; Rosenbaum et al., 2010).

In contrast to studies investigating the fundamentals of a basic step, most studies on variations of stepping movements that are required for behavioral flexibility have been limited to observations of limb kinematics. Particularly for turning, kinematics studies have shown, that it comes along with leg-specific changes in the tarsal placement, leg excursion angles and even stepping direction along the two body sides of the animal (Jindrich and Full, 1999; Dürr and Ebeling, 2005; Frantsevich and Cruse, 2005; Mu and Ritzmann, 2005; Gruhn et al., 2009; Bender et al., 2011; Dallmann et al., 2016; Dürr et al., 2018; Deangelis et al., 2019; Pfeffer et al., 2019). Using setups with a slippery walking surface for the stick insect (Gruhn et al., 2006; Gruhn et al., 2009) has helped demonstrating that these turning-related leg-specific kinematics largely result from the motor output to each individual leg, and do not depend on mechanical coupling between the legs or even on the presence of neighboring legs. This implies that the observed leg-specific kinematics must directly emerge from nervous system output.

Only recently, we have learned about certain changes in neural activity, both at the level of the premotor and motor networks, which may be responsible for the observed turning-related changes in kinematics: extracellular recordings and stimulation of the central complex in the cockroach, as well as by the identification of single neurons in *Drosophila* have shown that the seemingly simple behavioral transition from straight walking to turning is initiated by changes in the descending drive from the central complex of the cerebral ganglion (Ridgel et al., 2007; Guo and Ritzmann, 2013; Martin et al., 2015; Bidaye et al., 2020). Work in the cockroach and the stick insect showed that turning also involves the modification of local reflexes in the front (Mu and Ritzmann, 2008) and middle legs (Hellekes et al., 2012). In addition, changes in the local processing of sensory load and movement feedback further downstream in the thoracic ganglia have been reported (Gruhn et al., 2016; Schmitz et al., 2019). Finally, optomotor-induced turning behavior affects local motoneuron activity in a body side-specific fashion, and CPGs of the thoraco-coxal joint appear to be involved in this (Gruhn et al., 2016). Depending on the role of the leg as an inside or outside leg with respect to the turning direction, these modifications of local reflexes or CPG activity can also occur independently from each other, as was recently shown for the middle leg in the turning stick insect (Gruhn et al., 2016; Schmitz et al., 2019). Apart from the findings in the stick insect mesothoracic thorax-coxa-(ThC)-joint (Gruhn et al., 2016), only a few examples for behavior-dependent modification of CPG activity are known from vertebrates such as turtles, where a single network is known to produce various behaviors such as swimming or scratching (Field and Stein, 1997a; Field and Stein, 1997b; Stein, 2018), or to regulate different speeds as found in zebrafish (Ausborn et al., 2012; Ampatzis et al., 2014).

In the stick insect, each joint of a leg is apparently controlled by an individual pattern generating network (Büschges et al., 1995; Bässler and Büschges, 1998; Bidaye et al., 2018; Mantziaris et al., 2020). The central coupling among CPGs is so weak that a so called “fictive coordinated locomotion” of all muscle groups of a leg is not observed in recordings of leg nerve activity in deafferented preparations (Dean, 1989; Büschges et al., 1995; Ludwar et al., 2005a; Borgmann et al., 2009; Mantziaris et al., 2017). The relative independence of pattern generators and the modulation of their activities are a hallmark for the generation of behavioral flexibility with the same network structures, and a differential regulation of the joint motor networks and CPGs is suggested by the above-mentioned turning kinematics data (Dürr and Ebeling 2005; Gruhn et al., 2009; Dürr et al., 2018).

Based on the turning-related changes in the motor activity of the stick insect middle leg ThC-joint and the observed differences in leg-specific kinematics during curve walking, we raise the question whether the side specificity is typical for each leg on one side of the body, and even for each joint of a leg. We also ask, whether these joint-specific changes in motor activity are mediated by CPG activity. To investigate these questions, we systematically recorded the motor neuron (MN) activity related to the three main leg joints of the stick insect meso- and metathoracic legs during inside and outside turning of the front legs in regular saline. We also studied the effect of the optical turning stimuli on contralateral motor activity in ganglia treated with the CPG-activating muscarinic agonist pilocarpine, in order to study whether the different local joint CPGs are involved in changes of joint-specific motor activity.

## Materials and Methods

All experiments were performed at room temperature (21°C–24°C) on adult female stick insects of the species *C. morosus* (Brunner von Wattenwyl, 1907) that were raised on blackberry leaves *ad libitum* and kept at a 12 h:12 h light dark cycle. The experiments were carried out on an air cushioned table (MICRO-g, TMC, Peabody, MA, United States) surrounded by a darkened Faraday cage.

### Preparation and Experimental Design

For all experiments, all legs except for the two front legs were amputated at mid-coxa (Fischer et al., 2001). The animal was fixed ventral side down with dental cement (two-component glue, Protemp II, ESPE, Seefeld, Germany) onto a foam platform, which was thinner than the width of the insect (3 mm × 5 mm × 100 mm, W × H × L) and was mounted on a brass tube. The head and front legs protruded from the front of the stick allowing their free movement. The rod was positioned above a 13.5 cm × 13.5 cm polished acrylic glass plate at a height of about 8–12 mm to ensure resting angles of the front leg FTi-joint of about 110°. The plate was covered with a lubricant composed of 95% glycerin and 5% water to create a slippery surface (Gruhn et al., 2006) which allows unrestricted leg movements of the stationary animal. Turning in different directions was elicited by a progressive striped pattern displayed on two curved LED screens in front of the animal. Stepping either occurred spontaneously or was elicited by briefly touching the abdomen with a paintbrush. Sequences recorded during stimulation were not analyzed. All stepping sequences were filmed from above at 75 frames per second.

EMG electrodes were placed approximately 1–2 mm apart in the proximal half of the *flexor tibiae* muscle of both front legs, by punching two small holes into the cuticle using stainless steel minuten pins (0.15 mm), and inserting the cut ends of two twisted copper wires (OD 40 µm). The wires were fixed by applying a small amount of dental cement at the insertion points. The dorsal side of the thorax was opened, the gut was moved aside, and connective tissue was carefully removed to expose the meso- and metathoracic ganglia and the respective leg nerves. The remaining leg stumps were mechanically immobilized with dental cement applied to the coxa. All mesothoracic and metathoracic nerve roots, except for the nerves on which electrodes were placed, were cut prior to the recording to exclude local sensory input (deafferentation), and the body cavity was filled with saline (Weidler and Diecke, 1969).

*Pro*- and *retractor coxae* MN activity was recorded from nerves nl2 and nl5. *Levator* and *depressor trochanteris* MN activity was recorded from C1 and C2 nerves. The C1 nerve contains the axons of a large number of LevTr MNs (9–11 MNs, 1 CI, and 3 DUM cells; Goldammer et al., 2012). The C2 nerve innervating the *depressor trochanteris muscle* contains only the three large axons of the fast *depressor trochanteris MN* (FDTr, largest amplitude), the slow *depressor trochanteris MN* (SDTr, medium sized amplitude), and the common inhibitor (CI) (Schmitz, 1986). *Extensor tibiae* MN activity was recorded from nerve nl3 (Marquart, 1940; Graham, 1985, Figure 1). For recordings of the *flexor tibiae* MN the femur was cut in the proximal third and the stump fixed at an angle of apx. 75° with respect to the body long axis. The *extensor* and *flexor tibiae* muscles control extension and flexion of the leg tibia about the FTi-joint. The ExtTi is innervated by only 3 MNs: the fast *extensor tibiae* (FETi, largest spike amplitude) the slow *extensor tibiae* (SETi, medium spike amplitude), and the CI1 (Godden, 1972; Bässler, 1977; Bässler and Storrer, 1980; Bässler and Wegner, 1983). The flexor, on the other hand, is innervated by small branches of the *ncr*. These branches contain axons of 8–25 MNs, 1–2 DUM cells, and the CI2 and CI3 (Goldammer et al., 2012) which cannot be individually distinguished from each other. The leg was opened dorsally, and the *extensor tibiae* muscle as well as its muscle apodeme were carefully removed to gain access to the *nervus cruris* (*ncr*) and its side branches innervating the flexor.

**FIGURE 1:**
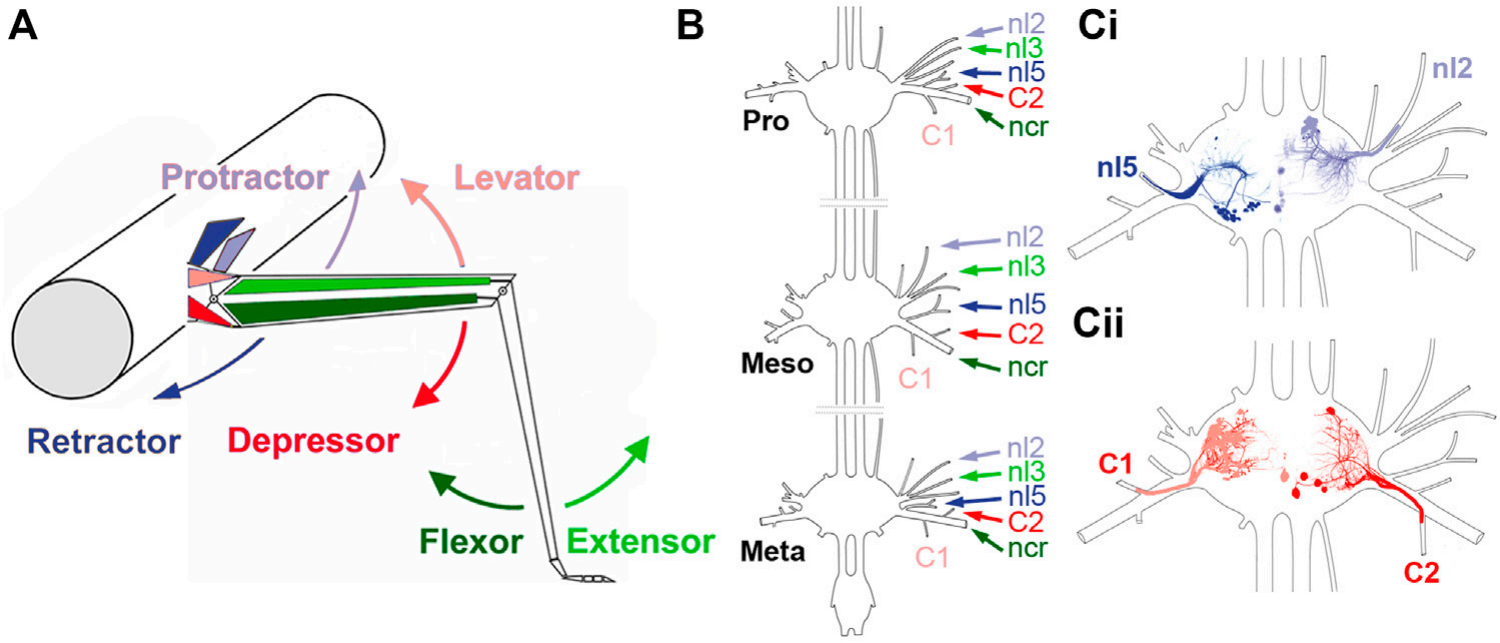
Summary of the stick insect leg segments, with an overview of the thoracic ganglia and relevant nerves. **(A)** Schematic representation of the middle leg and the mesothoracic body-segment, with the location of the muscles responsible for the movement direction that is depicted by the arrows. **(B)** Sketch of the three thoracic ganglia in the stick insect and the location of the relevant leg nerves that are being recorded in this study (Pro: prothorax, Meso: mesothorax; Meta: metathorax). **(Ci,Cii)** Sketches of a mesothoracic ganglion, showing the location of motor neurons of *protractor* (nl2) and *retractor coxae* (nl5) motor neurons **(Ci)**, and *levator* (C1) and *depressor trochanteris* (C2) that project through the respective lateral leg nerves.

Split-bath experiments were generally conducted as described in Borgmann et al. (2007), Borgmann et al. (2009) on either the mesothoracic, the metathoracic or both ganglia, being left interconnected in all cases. In brief, the body cavity was fixed with 0.2 mm stainless steel minuten pins, and a 5 mm piece of the cuticle anterior to the attachment points of the retractor muscle, and posterior to the target ganglion was removed, leaving only the anterior and posterior connectives, and the largest tracheae intact. We transiently removed saline from the body cavity, to build a vaseline (white Vaseline, Bad Apotheke, Bad Rothenfelde, Germany) rim separating the meso- and metathoracic body cavities. Care was taken to seal the area around the connectives and tracheae. At the end, the separated areas of the body cavity were refilled with saline and checked for leaks. After control experiments conducted in *Carausius* saline, saline was replaced in the examined ganglion compartment with a 3 mM pilocarpine solution in saline (Büschges et al., 1995).

### Electrophysiology

For EMGs of the *flexor tibiae* muscles of front legs, the electrodes were placed as described above. Nerve activity was recorded extracellularly from the nerves specified above using monopolar hook electrodes (modified after Schmitz et al., 1988). The signal was pre-amplified 100-fold (electronics workshop, Zoological Institute, Cologne, Germany), band-pass filtered (100–2,000 Hz), when necessary further amplified 10–1,000-fold, adapted to the signal-to-noise ratio. A reference electrode was placed in the abdomen of the animal. MNs were easily identifiable, as the investigated MNs had axons with the largest diameter in their respective leg nerve and therefore showed the largest amplitudes in the extracellular recordings (identification of MNs based on AP amplitude: Pearson et al., 1970; nl3: Bässler and Storrer, 1980; further information: nl2, nl5: Graham and Wendler, 1981; all leg nerves: Goldammer et al., 2012, Figure 1).

For intracellular recordings, the mesothoracic ganglion was stabilized using cactus spines on a ganglion holder covered with wax. The ganglion sheath was treated with proteolytic enzyme (Pronase E, Merck, Germany) for around 30 s. Intracellular recordings were performed using sharp glass microelectrodes (filled with 3 M KAc/0.1M KCl; R 15–25 MΩ). The electrodes were pulled with the Sutter Microelectrode puller (P-1000, Sutter Instruments, Novato, CA, United States). The intracellular signals were recorded in bridge mode, and amplified with an intracellular amplifier (SEC-10L, npi electronics, Tamm, Germany).

All electrophysiological signals were digitized using the MICRO 1401 II analog-digital converter (CED, Cambridge, United Kingdom) and recorded with the data acquisition and analysis software Spike2 (version 7.01, CED, Cambridge, MA, United Kingdom) on a personal computer running Windows 7 (Microsoft, Corporation, Redmond, WA, United States). To determine the stepping direction of the front legs, video files were synchronized with the respective Spike2 recordings, using a MATLAB (R2011b) script (kindly provided by Dr. Till Bockemühl).

### Data Analysis

Spiking activity of phasically active units, and the onset of *flexor tibiae* activity during front leg stepping were marked and saved in separate event channels (minimum interval: 3 ms) for each turning direction. The time series of the units were extracted by defining a threshold crossing through them which excluded common inhibitor (CI) spikes and spikes related to ventilatory activity that was synchronously active in all nerves (Graham and Wendler, 1981). Common inhibitor activity was easily discernible through its presence in multiple nerve recordings. As bursts, we defined bouts of spike activity with simultaneous cessation of activity in the nerve innervating the respective antagonist.

Data were first analyzed with respect to the step cycle of the front leg, which corresponds to the period between adjacent onsets of *flexor tibiae* muscle activity. Phase histograms with a bin size of 30° (i.e., 12 bins) were used to show the distribution of motoneuronal activity in the step cycle in each recording. The bin with the largest number of spike events indicates the phase of the FL step cycle at which motor neurons were maximally active (see Circular Statistics, next paragraph). The same approach was used for comparing activity between motor neuron pools on the inside and outside with respect to the turning direction.

Circular statistics were used to relate the neuronal activity to the step cycle of the ipsilateral front leg. The start of front leg flexor activity was defined by the beginning of the stance phase (ground contact), because of their tight correlation (Gruhn et al., 2006; Rosenbaum et al., 2010). MN activity was often not uniform throughout each step cycle, and polar plots were used to display the angle of maximal neuronal activity (see above). The Hodges-Ajne test was used to detect general deviations from uniformity (MATLAB toolbox). The mean vectors of the maxima, including their lengths, were computed *via* the MATLAB toolbox for circular statistics (Berens, 2009). Data from the intracellular recordings were tested for normal distribution with the Shapiro-Wilk test. Based on the result of the normal distribution test, the data were further analyzed with non-parametric tests at a significance threshold of *p* = 0.05 (Kruskal–Wallis test and the Dunn´s Multiple Comparison Test). For the Cross-correlation analysis the time series of the rectified and smoothed (time constant of 0.07 s) protractor, retractor, levator and depressor MN activity were exported with a sampling rate of 1,000 Hz and the signals were cross-correlated using a custom-written script in Matlab. The time constant used for smoothing ensured a good representation of the signals. An exaggeration of large-amplitude action potentials was not observed. All signals were smoothed 100-fold and their z-score was calculated before cross-correlating the full length of the recording intervals illustrated in each figure. Cross-correlations were calculated using the “xcorr” Matlab function after normalizing each data sequence, so that the autocorrelations at zero time lag are equal with 1, according to the formula:

$${\hat{R}}_{xy,coeff}\left(m\right)=\frac{1}{\sqrt{{\hat{R}}_{xx}\left(0\right){\hat{R}}_{yy}\left(0\right)}}{\hat{R}}_{xy}\left(m\right)$$

**FIGURE 2:**
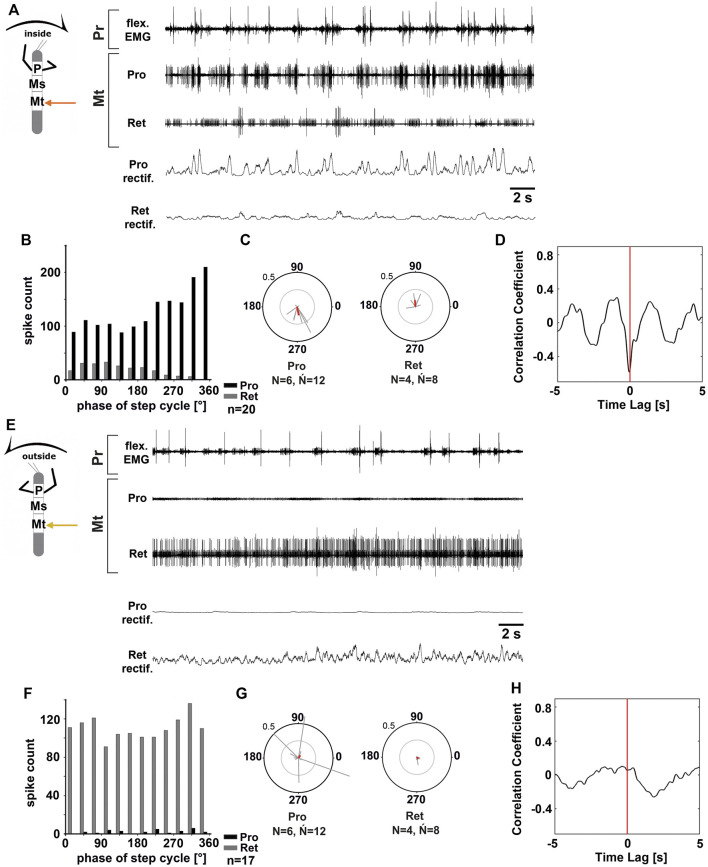
Motor output of the metathoracic *protractor* and *retractor coxae* MN pools in the deafferented ganglion during front leg (FL) inside **(A–D)** and outside **(F,H)** stepping of the same animal. **(A)** FL flexor EMG recording (top), together with extracellular recordings of the ipsilateral protractor (nl2 nerve, second trace) and retractor (nl5 nerve, third trace) nerve during an inside stepping sequence, as well as the rectified protractor and retractor activities (fourth and fifth trace); **(B)** Phase histogram of protractor and retractor nerve activity from the experiment in **(A)** with respect to the FL step cycle; **(C)** Polar plots with the spike event maxima (grey vectors) and their mean (red) for the metathoracic protractor (left) and retractor (right) with respect to the FL step cycle; **(D)** cross-correlation analysis of the protractor and retractor activity. **(E)** FL flexor EMG (top), together with extracellular recordings of the ipsilateral protractor (second trace) and retractor (third trace) nerve during an outside stepping sequence, as well as the rectified protractor and retractor activities (fourth and fifth trace); **(F)** Phase histogram of protractor and retractor nerve activity from the experiment in **(E)** with respect to the FL step cycle; **(G)** Polar plots with the spike event maxima (grey vectors) and their mean (red) leg for the metathoracic protractor (left) and retractor (right) with respect to the FL step cycle; **(H)** cross-correlation analysis of the protractor and retractor activity. *N* = number of animals; *Ń* = number of hemiganglia; *n* = number of analyzed steps. Pr, Mt: Pro- and Metathoracic location of the recording, respectively; Pro, Ret: *protractor coxae* and *retractor coxae* motor neuron activity from nerves nl2 and nl5, respectively. The sketch indicates a stick insect with front legs in a flexed (inside) or stretched (outside) position, and the arrow above indicating the turning direction, the arrow on the side the recorded thoracic segment.

The correlograms depict the correlation coefficient in the time lag window (−5 s, 5 s) (Figure 2). Between stepping sequences, pro- and retractor MNs are tonically active (Büschges and Schmitz, 1991). FL stepping frequency during turning sequences was either compared to pilocarpine-activated burst frequencies of the motor neuron pools residing on the inside and outside relative to the turning direction, or compared to the pilocarpine burst onsets in the meso- or metathoracic nerves under pilocarpine when the animal was quiescent.

The number of animals is represented by “N”, and the number of analyzed hemiganglia by “Ń”, as in many animal preparations leg nerves of both hemiganglia were recorded simultaneously. Thus, “Ń” corresponds to the actual number of experiments. The letter “n” corresponds to the number of FL steps analyzed in phase histograms.

## Results

When intact stick insects walk in a curved path, leg kinematics drastically change (Dürr and Ebeling, 2005; Gruhn et al., 2006; Gruhn et al., 2009). Apart from the drastic changes in protractor and retractor activity, recording EMG activity in the middle leg of the turning animal revealed only minor changes in strength of *flexor tibiae* and *depressor trochanteris* muscle activity, when comparing straight walking to inside or outside steps, respectively (Gruhn et al., 2006; Rosenbaum et al., 2010; Gruhn et al., 2011; Rosenbaum et al., 2015). Therefore, we first asked whether the observed changes in the motor output concern only premotor networks controlling the ThC-joint, or whether similar mechanisms are also active in the premotor networks that control the more distal CTr- and FTi-joints. For this purpose, we used a reduced preparation with all legs removed except the two front legs, that performed curved walking on a slippery surface due to an optomotor stimulation. At the same time, we recorded extracellularly from the deafferented meso- and metathoracic leg nerves nl2 (innervates *protractor coxae, ProCx*), nl5 (*retractor coxae, RetCx*), C1 (*levator trochanteris, LevTr*), C2 (*depressor trochanteris, DepTr*), nl3 (*extensor tibiae, ExtTi*) and from branches of nervus cruris (ncr) innervating the *flexor tibiae* (*FlxTi*; for a summary of the leg anatomy and innervating nerves, Figure 1), and recorded the activity from above with video to monitor the walking direction. For simplicity reasons, we call the recording site that is on the side of the inside stepping front leg “inside”, and that of the outside stepping front leg “outside”.

### Protractor and Retractor Coxae in the Meso- and Metathorax

Gruhn et al. (2016) previously showed a turning direction-dependent change in the strength of activity of mesothoracic ProCx and RetCx MN pools on the respective inside and outside. In addition, similar to findings by Borgmann et al. (2007) for treadwheel stepping, the mesothoracic inside MN pools showed alternating activity coupled to the FL steps, while no such rhythmicity was present on the outside (Gruhn et al., 2016). Moreover, Borgmann et al. (2007) showed that FL stepping has a weaker influence on the meta- than on the mesothoracic ThC-MN activity. All these findings considered, we therefore tested if there is also a difference in the turning-related motor output between the metathoracic ThC-joints.

Figure 2 shows the typical activity in the metathoracic nl2 (protractor) and nl5 (retractor) nerves during inside (Figures 2A–D) and outside steps (Figures 2E–H) of the ipsilateral front legs. The results in the metathoracic ThC-MN pools are very similar to those in the mesothorax during the respective behavior of the front legs for both sides.

On the inside, MN activity in metathoracic ProCx MNs was strong, while RetCx MN activity was weak (*N* = 6, *Ń* = 12) (Figure 2A). ProCx MN activity was coupled to the front leg steps with a mean vector pointing towards 265° (*N* = 6, *Ń* = 12; 11 out of 12 experiments, Figures 2B,C), while RetCx MN activity showed a preferred coupling to FL stepping in only four out of 12 experiments (average peak at around 90°, Figure 2C). On the outside, activity in the metathoracic RetCx MNs increased (Figure 2E) and was usually strongly tonic, while activity in the ProCx MNs was strongly decreased compared to the activity in the same nerve when recorded on the inside. In 50% of the experiments, RetCx MN activity showed occasional coupling to the front leg steps, but without a consistent phase relationship (Figures 2F,G), and maximal ProCx MN activity never showed a consistent phase relationship to the front leg steps (Figures 2F,G). Cross-correlation analysis confirmed the alternation between metathoracic ProCx and RetCx MN activity when recorded on the inside, whereas no such coupling was detected when recorded on the outside (Figures 2D,H).

In summary, during front leg turning steps, the metathoracic ProCx and RetCx MNs were activated in a similar manner as the respective mesothoracic MN pools. ProCx MNs were strongly activated and mostly rhythmic on the inside, whereas their activity decreased on the outside. At the same time, RetCx MN activity was strongly increased over ProCx MN activity on the outside, and not systematically coupled to the front leg steps.

### Levator and Depressor Trochanteris in the Meso- and Metathorax

The observed changes in the motor output of the left and right side of the body during curve walking, but not between the meso- and metathorax may suggest a similar control mechanism for the ThC joints among thoracic ganglia. However, whether a similar activation pattern also applies to the other leg joint MN pools remained unclear. We therefore investigated the motor output that drives the next more distal leg joint, the coxa-trochanter (CTr)-joint, which allows the leg to be lifted by the *levator trochanteris* muscle during leg swing and be depressed towards the substrate by the *depressor trochanteris* muscle during leg stance. The two muscles are innervated by the meso- and metathoracic leg nerves C1 and C2, respectively.

**FIGURE 3:**
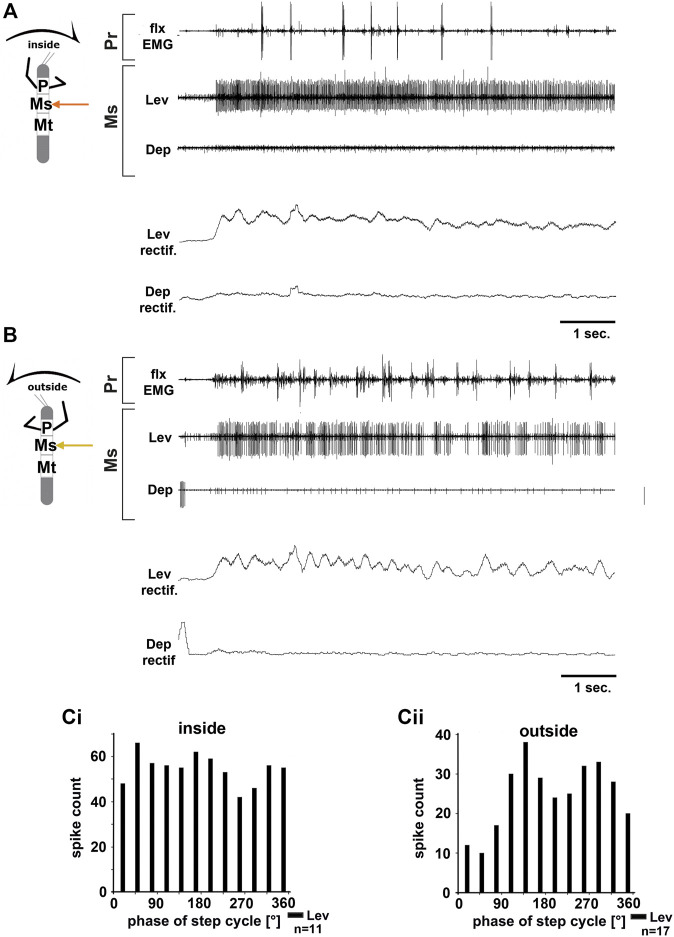
Motor output of the mesothoracic *levator* and *depressor trochanteris* MN pools in the deafferented ganglion during front leg (FL) inside **(A,Ci)** and outside **(B,Cii)** stepping from the same animal. **(A)** FL flexor EMG recording (top), together with extracellular recordings of the ipsilateral levator (C1 nerve, second trace) and depressor (C2 nerve, third trace) nerve during an inside stepping sequence, and the respective rectified activities (fourth and fifth trace); **(B)** FL flexor EMG (top), together with extracellular recordings of the ipsilateral levator (second trace) and depressor (third trace) nerve during an outside stepping sequence, and the respective rectified activities (fourth and fifth trace); **(Ci)** Phase histogram of levator and depressor nerve activity from the experiment in **(A)** with respect to the FL step cycle during inside stepping; **(Cii)** Phase histogram of levator and depressor nerve activity from the experiment in **(B)** with respect to the ipsilateral FL step cycle during outside stepping. *n* = number of analyzed steps. Pr, Ms: Pro- and Mesothoracic location of the recording, respectively; Lev, Dep: *levator trochanteris* and *depressor trochanteris* motor neuron activity from nerves C1 and C2, respectively. The sketch indicates a stick insect with front legs in a flexed (inside) or stretched (outside) position, and the arrow above indicating the turning direction, the arrow on the side the recorded thoracic segment.

In all animals, the change in mesothoracic C1 and C2 nerve activity upon font leg stepping was independent of the turning direction. LevTr MN activity on both the inside and the outside increased drastically and persisted throughout the entire walking sequence (*N* = 4, *Ń* = 6), whereas any spontaneous SDTr MN activity in C2 seized with the beginning of the walking sequence, and only common inhibitor (CI) activity was observed in these recordings (*N* = 3, *Ń* = 6, Figures 3A,B). No cross-correlation analysis was performed due to the lack of DepTr MN activity in the mesothoracic C2 nerve during FL turning steps. Occasionally, a modulation in the LevTr MN activity during FL steps is apparent, however, without a preferred phase with respect to the front leg (Figures 3B,Ci,Cii).

**FIGURE 4:**
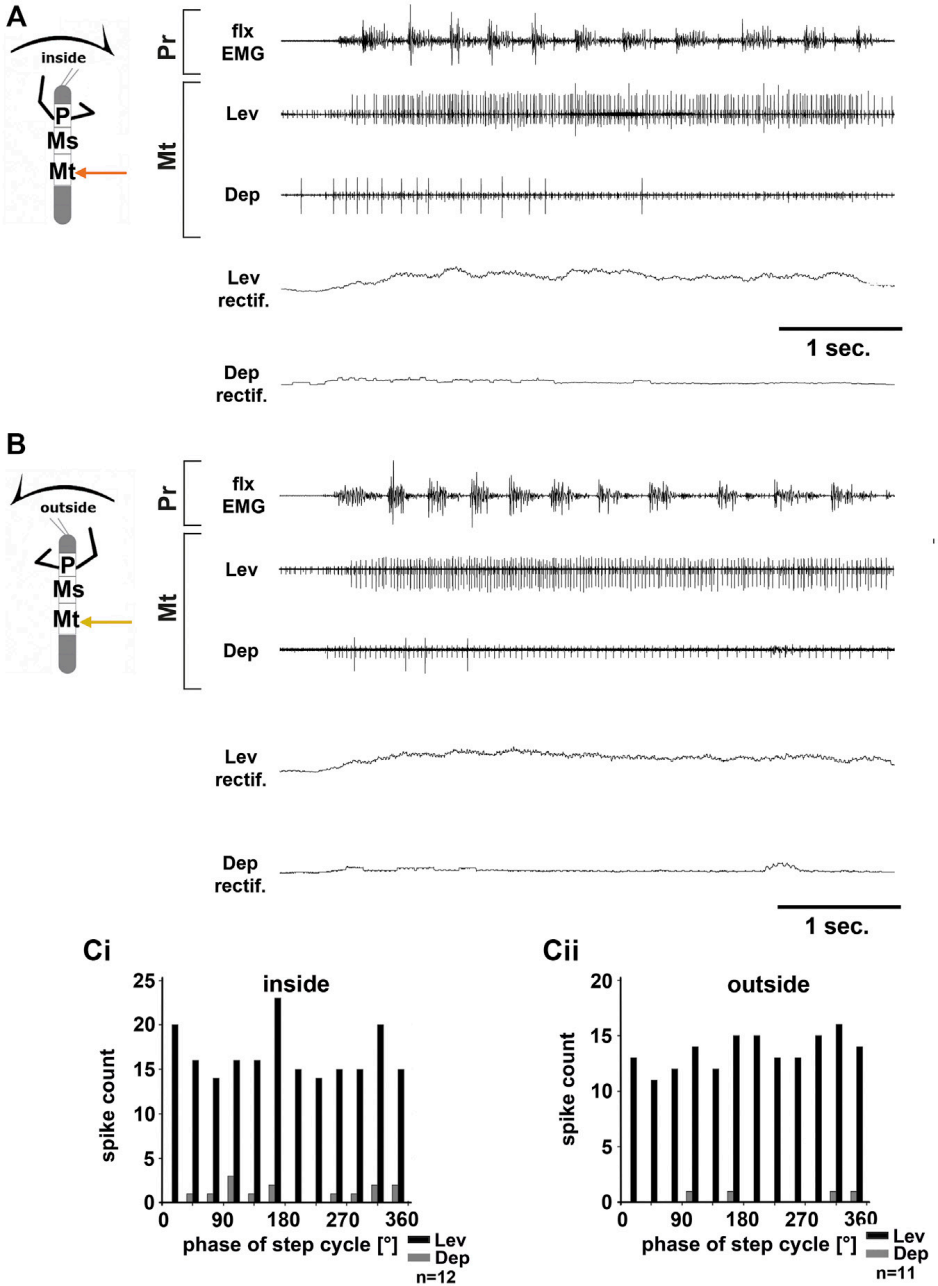
Motor output of the metathoracic *levator* and *depressor trochanteris* MN pools in the deafferented ganglion during front leg (FL) inside **(A,Ci)** and outside **(B,Cii)** stepping from the same animal. **(A)** FL flexor EMG recording (top), together with extracellular recordings of the ipsilateral levator (C1 nerve, second trace) and depressor (C2 nerve, third trace) nerve during an inside stepping sequence, and the respective rectified activities (fourth and fifth trace); **(B)** FL flexor EMG (top), together with extracellular recordings of the ipsilateral levator (second trace) and depressor (third trace) nerve during an outside stepping sequence, and the respective rectified activities (fourth and fifth trace); **(Ci)** Phase histogram of levator and depressor nerve activity from the experiment in **(A)** with respect to the FL step cycle during inside stepping; **(Cii)** Phase histogram of levator and depressor nerve activity from the experiment in **(B)** with respect to the ipsilateral FL step cycle during outside stepping. *n* = number of analyzed steps. Pr, Mt: Pro- and Metathoracic location of the recording, respectively; Lev, Dep: *levator trochanteris* and *depressor trochanteris* motor neuron activity from nerves C1 and C2, respectively. The sketch indicates a stick insect with front legs in a flexed (inside) or stretched (outside) position, and the arrow above indicating the turning direction, the arrow on the side the recorded thoracic segment.

Unlike in the ThC-joint, no turning-specific difference in MN activity was seen in the CTr-joint. Thus, we wanted to know whether differences in the control of the CTr-joint existed between the meso- and metathorax. Using a similar preparation, we recorded from metathoracic nerves C1 and C2 during front leg curve walking. Again, independent of walking direction, LevTr MN activity was drastically increased, and terminated as soon as front leg stepping ended (*N* = 6, *Ń* = 6). Figures 4A,B give examples for metathoracic C1 and C2 activity during front leg inside (Figure 4A) and outside steps (Figure 4B). In contrast to the mesothoracic C2 recording, we observed SDTr MN activity in the metathoracic C2 recording in 50% of the experiments with inside turns (*N* = 3, *Ń* = 3) and during 83% of the experiments with outside turns (*N* = 6, *Ń* = 5). Still DepTr MN activity was so scarce that no cross-correlation analysis was performed, and. despite occasional modulation of the LevTr MN activity, no phase coupling with respect to the ipsilateral front leg steps (Figures 4Ci,Cii) was detected.

In summary, front leg curve stepping sequences, initiate strong meso- and metathoracic LevTr MN activity, independent of the turning direction, and without phase coupling to the ipsilateral front leg step cycle. At the same time, activity of the mesothoracic DepTr MNs ceases or is greatly reduced in the metathorax.

### Extensor and Flexor Tibiae in the Meso- and Metathorax

Recording the motor activity of the two proximal leg joints in the inside and outside turning FL preparation revealed fundamental differences in the motor control between the two joints, body sides, and partially even between ganglia. Thus, we next sought to investigate, whether each leg joint is indeed subject to individual control mechanisms, or whether only one of the joints is controlled in a turning specific manner. For this, we recorded the activity of the motor neuron pools that control the FTi-joints of the meso- and metathoracic segments.

**FIGURE 5:**
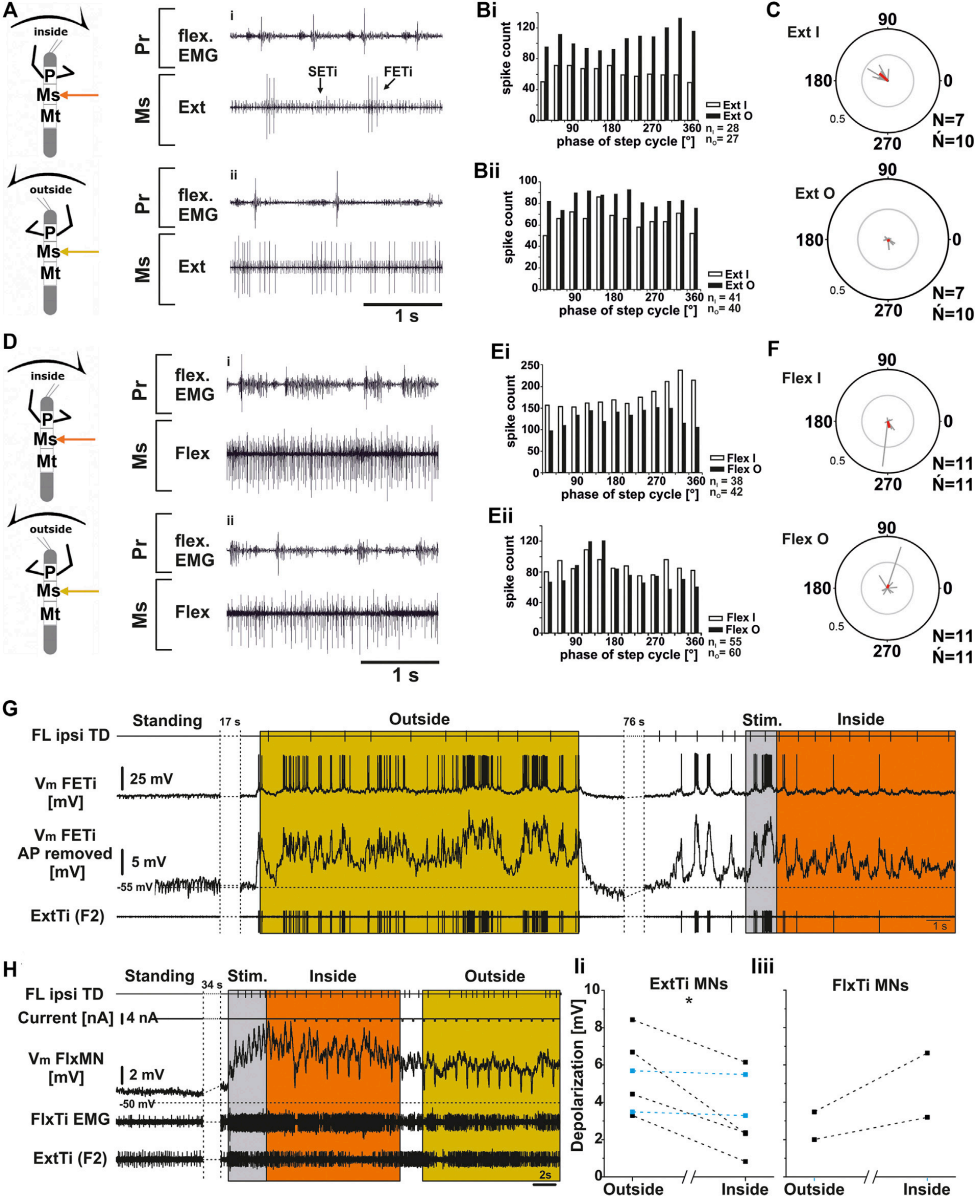
Mesothoracic *extensor* **(A–C)**; **(G,Ii)** and *flexor tibiae* **(D–F; H,Iii)** MN output in the deafferented ganglion during front leg (FL) inside and outside stepping. **(A)** flexor EMG of the front leg (top trace and third trace), together with extracellular recordings of the ipsilateral mesothoracic extensor nerve (nl3) during inside (i) and outside (ii) stepping sequences; **(Bi,Bii)**. Phase histograms of extensor nerve activity from the experiment shown in A **(Bi)**, and an additional experiment to show variability between experiments **(Bii)**, comparing inside and outside activity in the same animal with respect to FL step cycle. **(C)** Polar plots with spike event maxima (grey vectors) and their mean (red) for the extensor with respect to FL step cycle during inside (top) and outside (bottom) stepping; **(D)** flexor EMG recording of the FL (top trace and third trace), together with extracellular recordings of the ipsilateral mesothoracic flexor nerve (ncr) during an exemplary inside (i) and outside (ii) stepping sequence; **(Ei,Eii)**. Phase histograms of flexor nerve activity from walking sequences in **(Di)**, and **(Dii) (Ei)**, and a from second experiment to show the variability, comparing inside and outside activity in the same animal with respect to FL step cycle. **(F)** Polar plots with spike event maxima (grey vectors) and their mean (red) for the flexor with respect FL step cycle during inside (top) and outside (bottom) stepping; **(G–I)**: Tonic depolarization in ExtTi and FlxTi MNs during outside and inside stepping. **(G)** V_m_ of a fast extensor tibiae MN [FTi, with APs (second trace) and with APs mathematically removed (third trace)] during outside (yellow) and inside (orange) steps of the ipsilateral front leg (FL, top trace). In both situations V_m_ is depolarized over V_rest_, but the depolarization during outside leg stepping is increased over that during inside stepping. An extracellular recording of the F2 nerve with the ExtTi MN is shown in the bottom trace, Stim. (grey) marks a phase of brush strokes to the abdomen. **(H)** Flexor tibiae (FlxTi) MN recording (third trace) shows a stronger increase in tonic depolarization during inside (orange) leg stepping over that during outside stepping (yellow, top trace). Second trace shows current injection for input resistance measurements. Fourth trace and bottom trace show FlxTi EMG and extracellular F2 nerve recording. **(I)** Comparison of the amount of tonic depolarization in **(Ii)** ExtTi MNs and **(Iii)** FlxTi MNs between outside and inside stepping; blue lines show experiments with no change; significance value: *p* < 0.05 (*). *Ń* = number of experiments, (∼) approximately equal activity between inside and outside; *N* = number animals; *Ń* = number of analyzed hemiganglia; *n* = number of analyzed steps. Pr, Ms: Pro- and Mesothoracic location of the recording resp.; Flex/FlxTi, Ext/ExtTi: *flexor tibiae* and *extensor tibiae* activity, respectively. FETi: *fast extensor tibiae* motor neuron; V_m_: membrane potential. The sketch indicates a stick insect with front legs in a flexed (inside) or stretched (outside) position, and the arrow above indicating the turning direction, the arrow on the side the recorded thoracic segment.

We first recorded mesothoracic extensor (nl3) activity during FL turning. Throughout the experiments, no consistent pattern of ExtTi MN activity during either inside or outside front leg steps was discernible. Within the nl3 recordings, SETi and FETi APs were readily distinguishable in all experiments (*N* = 7, *Ń* = 10). There was always spontaneous SETi activity, whereas FETi activity appeared only with the begin of a stepping sequence. In all experiments outside ExtTi MN activity was greater than that on the inside in most walking sequences (Figures 5Ai,Bi). However, in five experiments there were also interspersed walking sequences with similar ExtTi MN activity between both sides (phase plot from another experiment in Figure 5Bii). Phase coupling of inside ExtTi MN activity to the front leg steps was observed in seven out of ten experiments (Figure 5C “Ext I”, mean phase around 140°), whereas outside ExtTi MN activity was phase coupled in only three out of 10 experiments (Figure 5C, “Ext O”).

Similarly, mesothoracic FlxTi MN activity, as recorded from the *ncr*, showed no consistent activity pattern (*N* = 11, *Ń* = 11). FlxTi MN activity was stronger during inside stepping sequences in nine out of 11 experiments. However, two animals showed no difference in activity throughout the experiment, irrespective of the walking direction, and in five of the other nine experiments, walking sequences with similar FlxTi MN activity between inside and outside stepping were equally observed. A representative example of original data is shown in Figures 5Di,ii, 5Ei for an experiment with stronger inside than outside activity, and in Figure 5Eii, there is an example showing similar FlxTi MN activity irrespective of the turning direction as phase histogram. FlxTi MN activity was never consistently coupled to the front leg step cycles (Figure 5F).

From previous work, it is known that motor neuron activity during stepping can be generated by a combination of tonic depolarizing drive as well as phasic excitation from sense organs, and phasic inhibition from the joint CPG (Büschges, 1998; Büschges et al., 2004; Ludwar et al., 2005b; see summary in Büschges and Schmidt, 2015). Currently it is unclear how the observed differences in MN activity during turning are produced. In a first attempt at elucidating the underlying mechanisms, we also recorded intracellularly from extensor and flexor MNs of the mesothoracic FT-joint (Figures 5G–I). We evaluated the depolarization of ExtTi and FlxTi MNs during induced turns and compared it to the resting membrane potential (V_m_) in the quiescent animal. We compared the V_m_ from six ExtTi MNs recorded during outside or inside stepping of the front legs in multiple walking sequences from six animals. During curve walking the V_m_ was always more depolarized compared to the resting animal. However, in four out of six animals and in the pooled data, the change in V_m_ of the ExtTi MNs from the inactive animal to that during outside stepping was greater than that during inside stepping (*p* < 0.05; Figures 5G,Ii). In the other two animals, the depolarization was the same between the two sides. The mesothoracic FlxTi MNs were equally depolarized upon stepping of the front legs as has been described before (Ludwar et al., 2005b; Westmark et al., 2009). However, the change in the V_m_ of the FlxTi MNs during turning of the front legs differed from the effect on ExtTi MNs, as the depolarization recorded during outside turns was smaller compared to that during inside turns (*N* = 2). These findings are in line with the changes observed in the extracellular recordings shown above in Figures 5A,D.

We also recorded the extracellular activity of ExtTi and FlxTi MNs in the metathoracic segment. Similar to the findings in the mesothoracic segment, we neither detected for the ExtTi, nor the FlxTi MNs a characteristic activity pattern, irrespective of the turning direction (Supplementary Figure S1). For the ExtTi MN, we found walking sequences in all experiments, during which the motor neuron activity was similar for both inside and outside MN pools (*N* = 4, *Ń* = 6). However, in all animals there were also walking sequences with higher ExtTi MN activity during outside than during inside FL stepping (*N* = 4, *Ń* = 5), whereas in two animals, walking sequences with the opposite effect were found (*N* = 2, *Ń* = 3). In contrast to the mesothoracic ExtTi MN activity, metathoracic ExtTi MN activity showed no preferred phase coupling to the front leg step cycle (Supplementary Figures 1A–C). Similar results were obtained for FlxTi MN activity (Supplementary Figures 1D–F). In all eight animals, walking sequences were found, where inside and outside FlxTi MN activity was the same. However, four animals showed walking sequences in which either the activity of the outside or the inside FlxTi MNs was greater during FL turning (*N* = 4, *Ń* = 4). Similar to the extensor recordings, no phase coupling of the activity to the front leg step cycle was detected on either side during turning.

In summary, for the mesothoracic ExtTi and FlxTi MNs recorded either on the inside or on the outside, no clear activity patterns were observed. However, outside ExtTi MN activity tends to be stronger compared to inside ExtTi MNs, whereas FlxTi MN activity shows the opposite effect, namely stronger activity on the inside in comparison to the outside. Similar to the protractor coxae MN activity, there is an, albeit weak phase coupling of the mesothoracic ExtTi MN activity to front leg step cycle. The concomitant changes in the V_m_ could suggest a task-specific change in the excitatory drive to the two antagonistic MN pools of the FTi-joint, which may be at least partly responsible for the observed changes in the motor output to this joint during turning. In the metathorax, there was also no specific activity pattern for the metathoracic ExtTi and FlxTi MNs observed irrespective of the FL turning direction. In addition, there was no phasic influence from the front legs.

### Involvement of Central Pattern Generators in the Control of Turning-Related Motor Output

Movement of each leg in the stick insect is known to be mediated by separate joint CPGs that control each of the three main leg joints (Büschges et al., 1995). Gruhn et al. (2016) could show that the specific changes in motor output during turning involved changes in CPG activity. Considering the weak coupling among CPGs that control the antagonistic joint MN pools within and between thoracic segments (Büschges et al., 1995; Mantziaris et al., 2017), and since the motor output of the MN pools showed clear joint- and thorax-segment-specific differences during turning, the question arose, whether CPGs are also involved in the observed changes in the other joints. We therefore used the split-bath approach from Borgmann et al. (2007) and Gruhn et al. (2016) in which the muscarinic ACh agonist pilocarpine (3 mM in stick insect saline) was applied selectively to either the meso- or the metathoracic ganglion, to initiate CPG activity. Subsequently, we elicited FL turning steps to investigate potential effects on the elicited rhythm.

### Metathoracic Thorax-Coxa Joint

First, we investigated the effect of FL turning on the pilocarpine-induced rhythmic output of the metathoracic ThC-joint CPG. Gruhn et al. (2016) reported for the respective mesothoracic CPG, that the pilocarpine rhythm on the inside speeds up with the onset of stepping, and that ProCx MN bursts had a similar phase relationship to the front leg steps as in the control without pilocarpine. In contrast, no change in the mean frequency of the pilocarpine rhythm but an increase in the strength of RetCx MN activity were reported.

**FIGURE 6:**
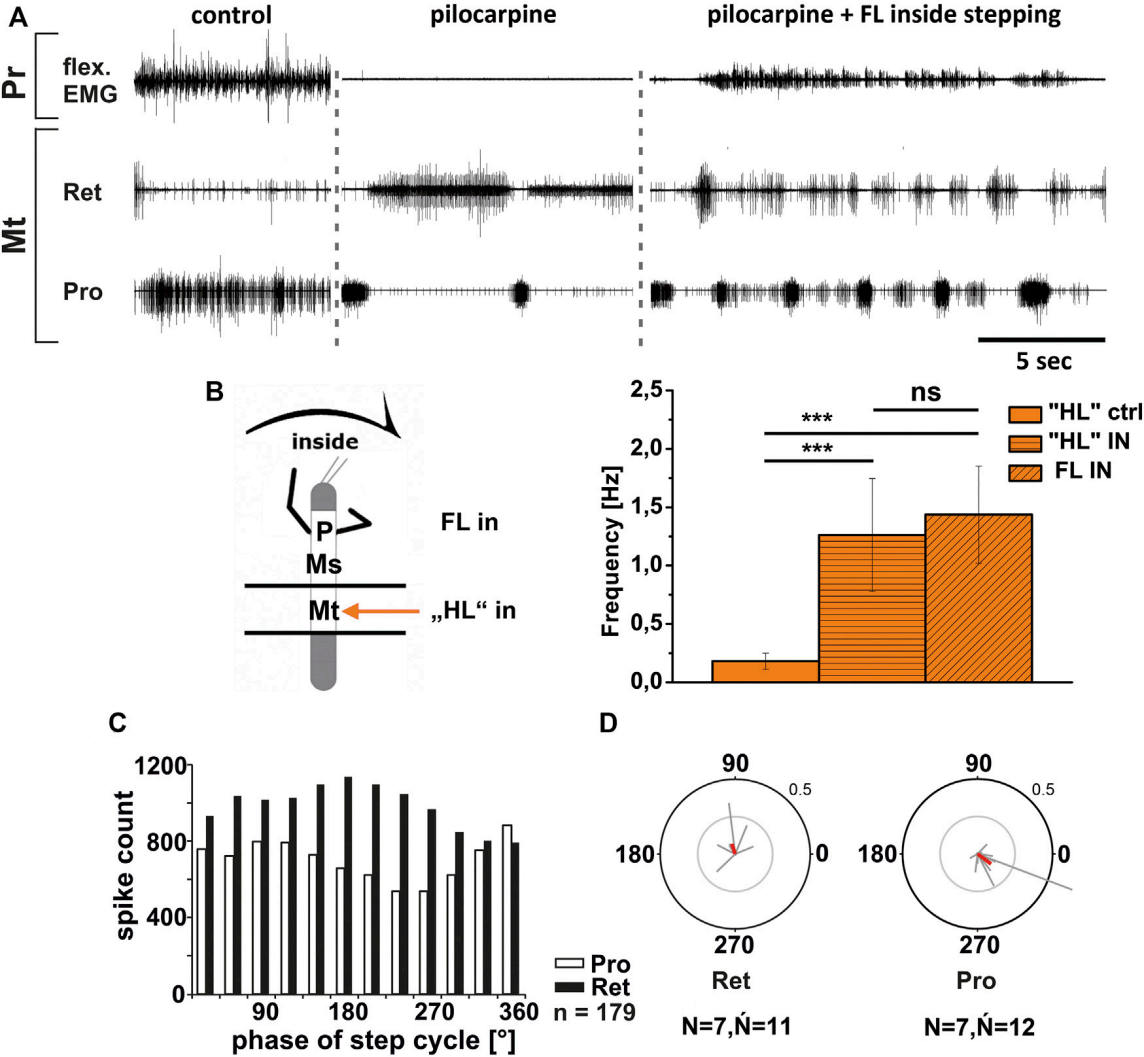
Motor output of *protractor* (Pro) and *retractor* (Ret) *coxae* MN pools in the pilocarpine activated deafferented metathoracic ganglion during front leg (FL) inside stepping; **(A)**. FL Flexor EMG (“flex”, top), together with extracellular recordings of the ipsilateral metathoracic retractor (nl5 nerve, second trace) and protractor (nl2 nerve, third trace) nerve during control inside steps, in pilocarpine w/o FL steps, and during inside stepping under pilocarpine application; **(B)**. Schematic of experimental split-bath configuration with comparison of nl2 pilocarpine control Burst frequency (“HL”ctrl) with deafferent HL-segment nl2 burst frequency (“HL”IN) during inside steps of the front legs (FL IN) from 21 walking sequences in 11 experiments (significance level <0.001) **(C)**. Phase histogram of retractor and protractor nerve activity under pilocarpine during inside stepping, with respect to the FL step cycle; **(D)**. Polar plots with the spike event maxima (grey vectors) and their means (red) for the metathoracic retractor (left) and protractor (right) during pilocarpine application with respect to the FL step cycle; *N* = number of animals; *Ń* = number of analyzed hemiganglia; *n* = number of analyzed steps. The sketch indicates a stick insect with front legs in a flexed (inside) or stretched (outside) position, and the arrow above indicating the turning direction, the arrow on the side the recorded thoracic segment. The black bars symbolize the vaseline barrier.

**FIGURE 7:**
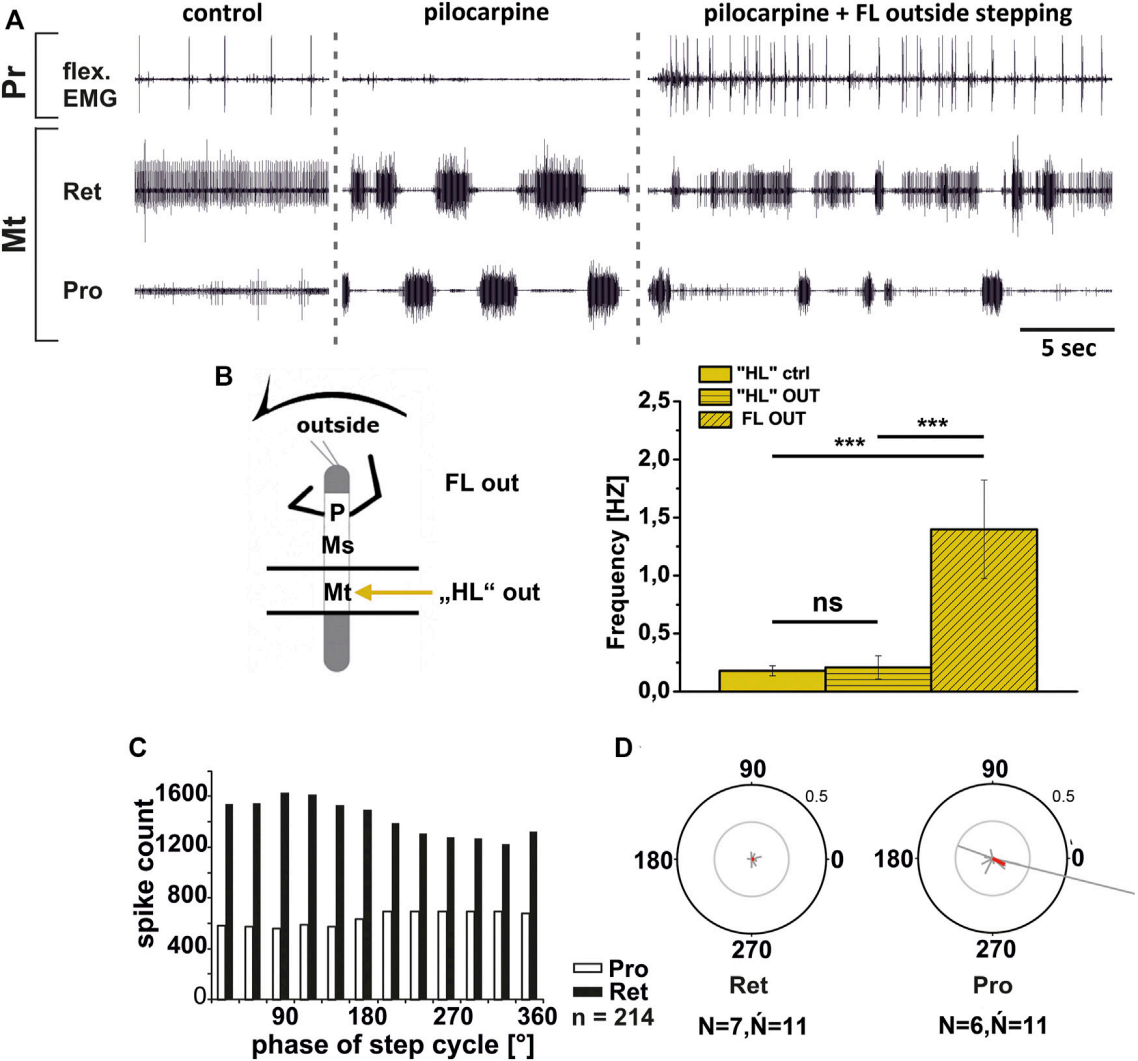
Motor output of *protractor* (Pro) and *retractor* (Ret) *coxae* MN pools in the pilocarpine activated deafferented metathoracic ganglion during front leg (FL) outside stepping; **(A)**. FL Flexor EMG (“flex”, top), and extracellular recordings of the ipsilateral metathoracic retractor (second trace) and protractor (third trace) nerve during control outside steps, in pilocarpine w/o FL steps, and during outside stepping under pilocarpine application; **(B)**. Schematic of experimental split-bath configuration with comparison of nl2 pilocarpine control Burst frequency (“HL”ctrl) with deafferent HL-segment nl2 burst frequency (“HL”OUT) during outside steps of the front legs (FL OUT) from 14 walking sequences in 10 experiments (significance level <0.001); **(C)**. Phase histogram of retractor and protractor nerve activity under pilocarpine during outside stepping, with respect to the FL step cycle; **(D)**. Polar plots with the spike event maxima (grey vectors) and their means (red) for the metathoracic retractor (left) and protractor (right) during pilocarpine application with respect to the FL step cycle; *N* = number of animals; *Ń* = number of analyzed hemiganglia; *n* = number of analyzed steps. Pr: Prothoracic/FL segment. The sketch indicates a stick insect with front legs in a flexed (inside) or stretched (outside) position, and the arrow above indicating the turning direction, the arrow on the side the recorded thoracic segment. The black bars symbolize the vaseline barrier.

Similar to these reported findings, metathoracic inside ProCx MN activity increased while RetCx MN activity decreased upon FL turning steps (Figure 6A). This was true independent of whether pilocarpine was applied only on the metathoracic ganglion or on the abdominal body cavity as well. Pilocarpine-induced bursting frequency increased from an average of 0.18 Hz (SD 0.07) to 1.26 Hz (SD 0.48), which was not significantly different from the respective FL stepping frequency (1.44 Hz, SD 0.42; Figure 6B). Inside ProCx MN activity was significantly coupled to FL steps in eight out of 12 experiments, with a mean phase of 300°, while inside RetCx MN activity had a preferred phase of 105° (six out of 11 experiments, *N* = 7, *Ń*_*Pro*_ = 12, *Ń*_*Ret*_ = 11; Figures 6C,D). Outside RetCx MN activity and RetCx MN bursts were stronger and often prolonged during FL turning compared to the pilocarpine rhythm in quiescence, similar to the changes observed in mesothoracic activity (Gruhn et al., 2016) (Figure 7A). However, the pilocarpine-induced average bursting frequency of the RetCx MNs in the quiescent animal (0.18 Hz, SD 0.043), and during outside FL steps (0.21 Hz, SD 0.01) did not differ significantly, while both were significantly slower than the stepping frequency of the ipsilateral FL (1.4 Hz, SD 0.42; Figure 7B). No systematic phase coupling to the outside FL step cycle for either the ProCx MNs (*N* = 6, *Ń* = 11) or the RetCx MN bursts was found (*N* = 7, *Ń* = 11), where only five out of 11 and three out of 11 experiments, respectively, showed significant coupling (Figures 7C,D).

In summary, the pharmacologically activated metathoracic ProCx and RetCx MN activity was clearly modified in both turning directions. Inside ProCx MN activity, and outside RetCx MN activity are stronger compared to the control bursts in the quiescent animal. In addition, on the inside and not on the outside, the pilocarpine rhythm shows significant increase in bursting frequency and phase coupling to the ipsilateral FL in most experiments. This effect is in line with the results in the mesothoracic ganglion, and indicates a change of local CPG activity in parallel with the turning direction of the front legs.

### Mesothoracic and Metathoracic Coxa-Trochanter Joint

**FIGURE 8:**
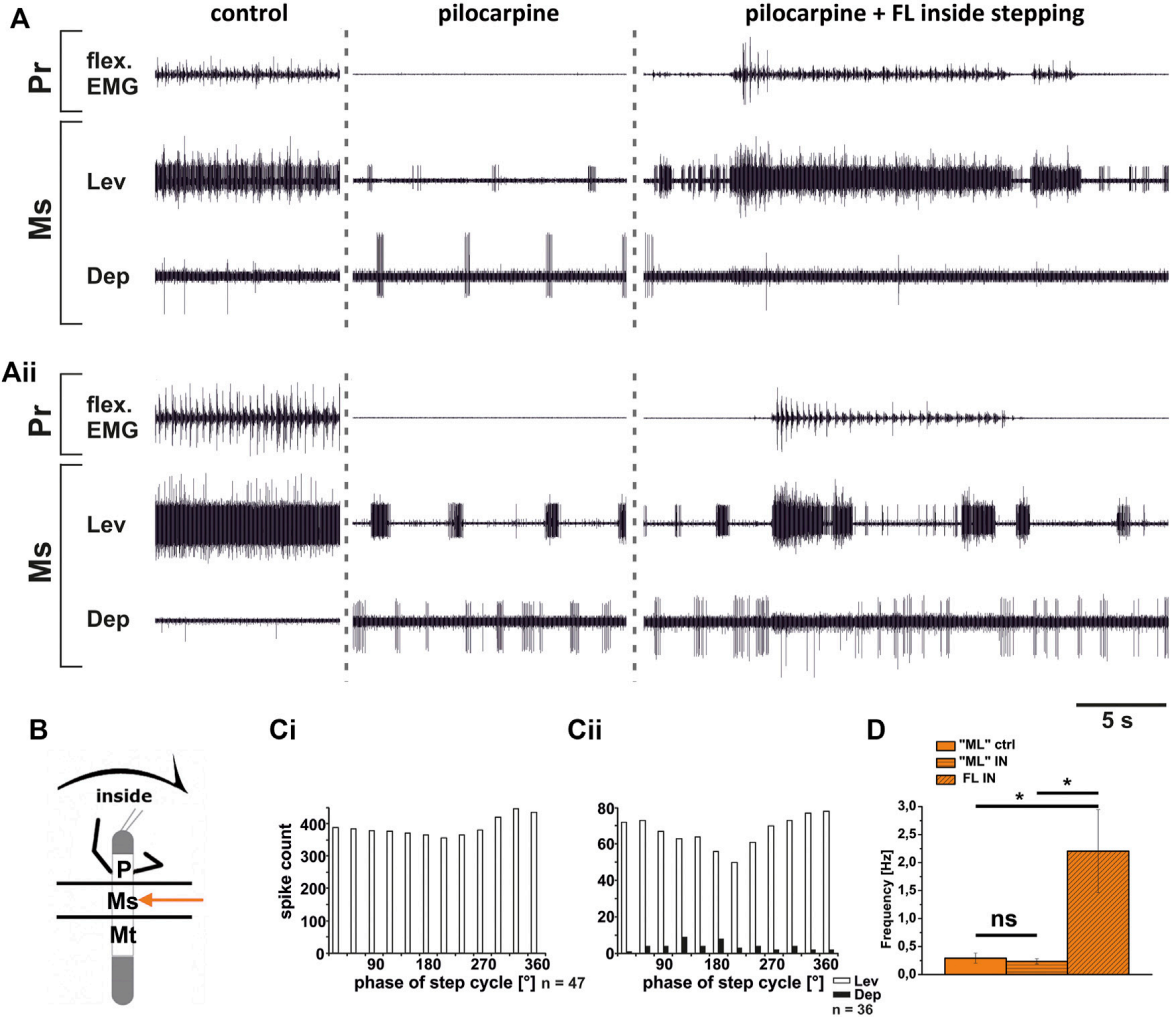
Motor output of *levator* (Lev) and *depressor* (Dep) *trochanteris* MN pools in the pilocarpine activated deafferented mesothoracic ganglion (Ms) during front leg (FL) inside stepping; **(Ai,Aii)** two examples for FL Flexor EMG (“flex”, top), together with extracellular recordings of the ipsilateral mesothoracic levator (C1 nerve, second trace) and depressor (C2 nerve, third trace) nerve during control inside steps, in pilocarpine w/o FL steps, and during inside stepping under pilocarpine application; **(B)**. Schematic of experimental split-bath configuration; **(Ci,Cii)** Phase histograms of levator and depressor nerve activity under pilocarpine during inside stepping from the animals in **(Ai,Aii)**, with respect to the FL step cycle; **(D)**. Comparison of pilocarpine C1 control Burst frequency (“ML”ctrl) with deafferent ML-segment C1 burst frequency (“ML”IN) during inside steps of the front legs (FL IN) from 18 walking sequences in three experiments (significance level <0.05); *n* = number of analyzed steps. Pr: prothoracic/FL segment. ns: not significant. The sketch indicates a stick insect with front legs in a flexed (inside) or stretched (outside) position, and the arrow above indicating the turning direction, the arrow on the side the recorded thoracic segment. The black bars symbolize the vaseline barrier.

**FIGURE 9:**
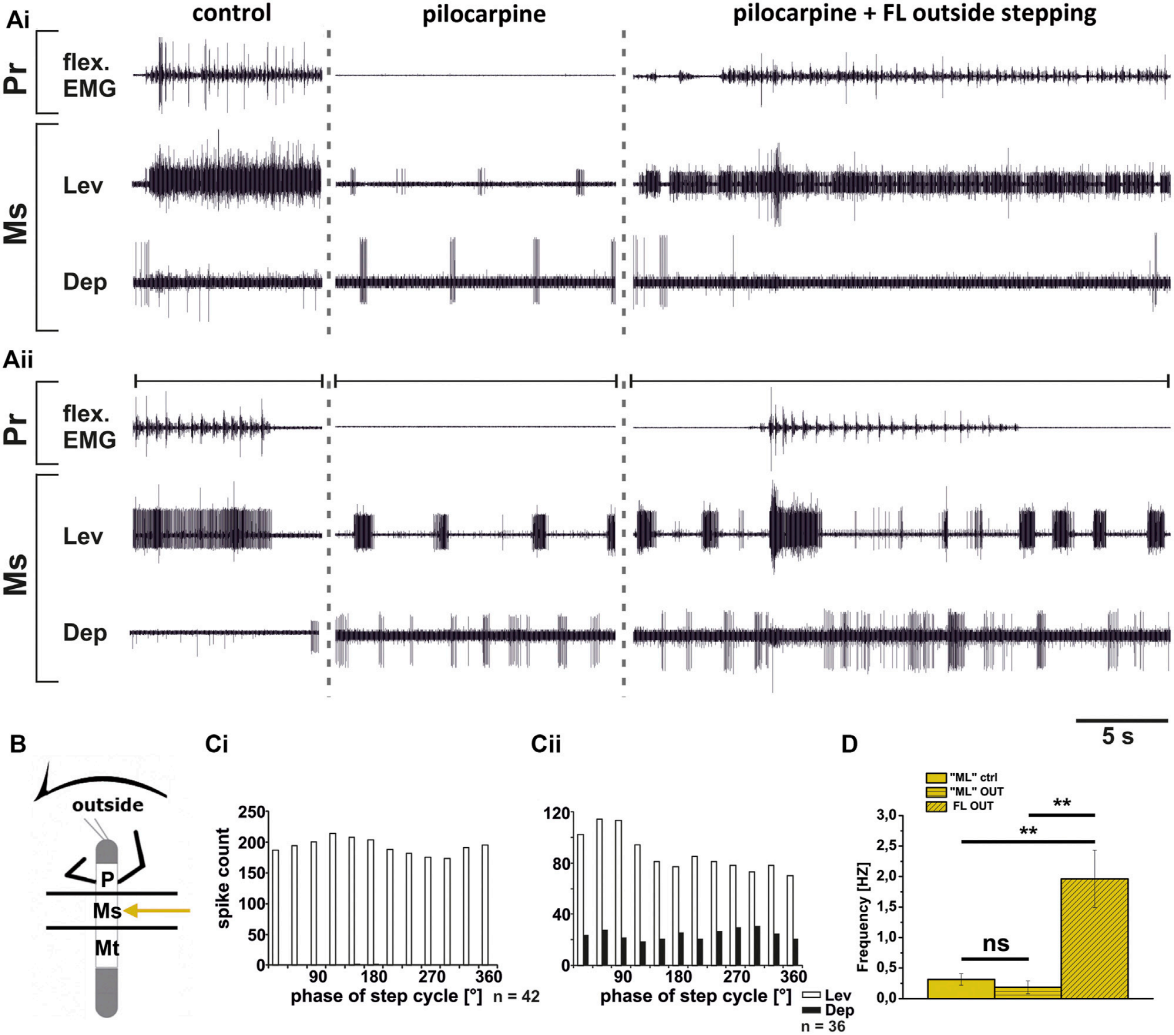
Motor output of *levator* (Lev) and depressor (Dep) *trochanteris* MN pools in the pilocarpine activated deafferented mesothoracic ganglion (Ms) during front leg (FL) outside stepping; **(Ai,Aii)** two examples for FL Flexor EMG (“flex”, top), together with extracellular recordings of the ipsilateral mesothoracic levator (C1 nerve, second trace) and depressor (C2 nerve, third trace) nerve during control outside steps, in pilocarpine w/o FL steps, and during outside stepping under pilocarpine application; **(B)**. Schematic of experimental split-bath configuration; **(Ci,Cii)**. Phase histograms of levator and depressor nerve activity under pilocarpine during outside stepping from the animals in **(Ai,Aii)**, with respect to the FL step cycle; **(D)**. Comparison of pilocarpine C1 control Burst frequency (“ML”ctrl) with deafferent ML-segment C1 burst frequency (“ML”OUT) during inside steps of the front legs (FL OUT) from 25 walking sequences in five experiments (significance level <0.01); *n* = number of analyzed steps. Pr: prothoracic/FL segment. ns: not significant. The sketch indicates a stick insect with front legs in a flexed (inside) or stretched (outside) position, and the arrow above indicating the turning direction, the arrow on the side the recorded thoracic segment. The black bars symbolize the vaseline barrier.

In contrast to the ThC-joint, no side-specific change in MN activity was detected in recordings of CTr-joint LevTr and DepTr MN pools during either inside or outside turning of the ipsilateral front legs in both the meso- and the metathoracic ganglion. However, given the weak coupling between joint CPGs, we asked, whether also this lack of side-specific activity is mediated through changes in CPG activity. After establishment of a pilocarpine-induced rhythmic alternation between mesothoracic LevTr and DepTr MN activity, front leg curve walking was induced. The results are summarized in Figures 8, 9 for inside and outside stepping, respectively. With the beginning of FL stepping and independent of the turning direction, LevTr MN bursts were prolonged and DepTr MN activity ceased entirely throughout the walking sequence in all experiments (*N* = 5, *Ń* = 14). Walking sequences with single APs in the C2 recording were observed in four experiments, and only rarely, occasional DepTr MN bursts during single walking sequences were observed (*N* = 2, *Ń* = 3, Figures 8Aii,Cii, 9Aii,Cii). Accordingly, no systematic coupling of either the inside or the outside LevTr and DepTr MN activity with respect to the ipsilateral FL step cycle was observed (Figures 8C, 9C). Despite the clear increase in LevTr MN, and decrease in DepTr MN activity, the pilocarpine-induced bursting frequency of 0.24 Hz (SD 0.05) during inside stepping sequences was on average unchanged compared to the control bursts with 0.29 Hz (SD 0.09). However, this was significantly different from the respective FL stepping frequency (2.2 Hz, SD 0.74; Figure 8D). A similar response was found in the outside turning animals, where the average control burst frequency (0.31 Hz, SD 0.09) was not significantly different from the bursting frequency of 0.18 Hz (SD 0.1) during FL turning even though the rhythm sometimes appeared to be locked in levator phase. Both bursting frequencies were significantly lower than the FL stepping frequency of 1.96 Hz (SD 0.47; Figure 9D).

**FIGURE 10:**
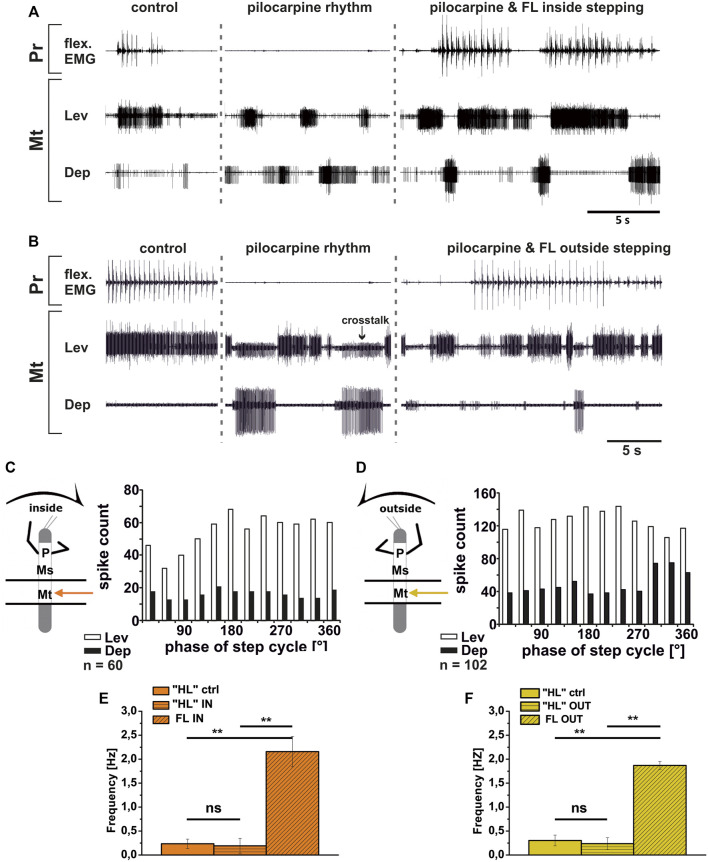
Motor output of *levator* (Lev) and *depressor* (Dep) *coxae* MN pools in the pilocarpine activated deafferented metathoracic ganglion (Mt) during front leg (FL) inside **(A,C)** and outside **(B,D)** stepping; **(A,B)**. FL Flexor EMG (“flex”, top), together with extracellular recordings of the ipsilateral metathoracic levator (C1 nerve, second trace) and depressor (C2 nerve, third trace) nerve during control inside **(A)** or outside **(B)** steps, in pilocarpine w/o FL steps, and during inside and outside stepping under pilocarpine application, respectively; **(C,D)**. Phase histograms of levator and depressor nerve activity under pilocarpine during inside **(C)** and outside **(D)** stepping, with respect to the FL step cycle; **(E,F)**. Comparison of pilocarpine C1 control Burst frequency (“HL”ctrl) with deafferent HL-segment C1 burst frequency (“HL”IN/OUT) during inside/outside steps of the front legs (FL IN/OUT) from 14 inside, and 7 outside walking sequences in 4, respectively Three experiments (significance level <0.01); *n* = number of analyzed steps. ns: not significant; Pr: prothoracic segment. The sketch indicates a stick insect with front legs in a flexed (inside) or stretched (outside) position, and the arrow above indicating the turning direction, the arrow on the side the recorded thoracic segment. The black bars symbolize the vaseline barrier.

Although DepTr MNs in the mesothorax hardly ever showed activity during control conditions, we observed occasional slow DepTr MN activity in the metathorax in about 50% of the experiments. We wanted to know whether this difference was also reflected in the changes in activity under pilocarpine influence. We therefore recorded the activity of the same 2 MN pools in the metathoracic ganglion after pilocarpine application and during FL curve stepping. The results are summarized in Figure 10. As in the mesothorax, in all experiments LevTr MN activity was stronger (*N* = 4, *Ń* = 7), and DepTr MN activity weaker (*N* = 3, *Ń* = 6) during turning sequences of the ipsilateral front legs, independent of the direction (Figures 10A,B). In all animals, sequences were observed, where DepTr MN activity was absent throughout front leg stepping. However, in five out of six experiments in which C2 activity was recorded, there were FL stepping sequences, during which intermittent bursts of DepTr MNs occurred (*N* = 3, *Ń* = 5). Again, no systematic coupling of either LevTr MN or DepTr MN activity with respect to the ipsilateral FL step cycle was observed, irrespective of the turning direction (Figures 10C,D). Similar to what was observed in the mesothorax, the respective control burst frequencies of 0.23 Hz (inside, SD 0.09), and 0.3 Hz (outside, SD 0.11) did not significantly differ from the frequencies of 0.19 Hz (inside, SD 0.15) and 0.24 Hz (outside, SD 0.12), while the FL stepping frequency was always significantly higher (inside: 2.16 Hz, SD 0.32; outside: 1.87 Hz, SD 0.08; Figures 10E,F) compared to the frequency of the MN rhythm.

In summary, coxa-trochanteral MN pools of the pilocarpine-activated meso- and metathorax showed a similar activation pattern as during control conditions without pilocarpine. This change of the regular, alternating pilocarpine rhythm to a clear increase in LevTr MN activation suggests that also at the level of this joint, CPGs are involved in the changes of the motor output in response to FL turning behavior. The occurrence of occasional DepTr MN bursts in the metathoracic C2 recording during stepping sequences suggests that the metathoracic CPG may be more weakly affected by FL turning than the mesothoracic CPG.

## Discussion

**FIGURE 11:**
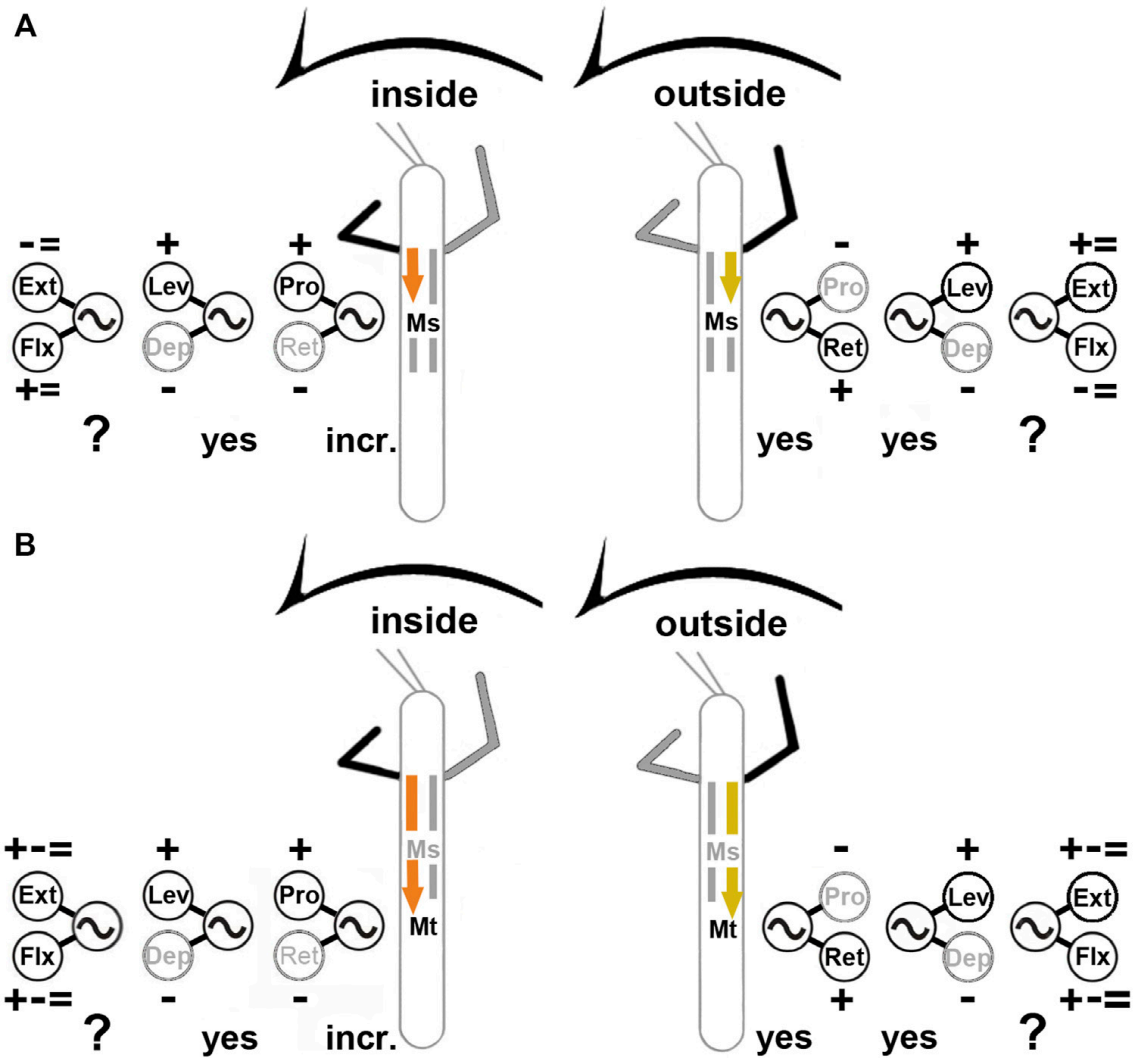
Summary of observed influences on the meso- **(A)** and metathoracic **(B)** MN pools during inside (left) and outside (right) turning of the front legs. “+”: increase in MN activity, “−”: decrease in MN activity, “ = ”: no change in MN activity found; “incr.”: increase in CPG frequency; “yes”**:** CPG affected; “?”: effect on CPG not tested. Ext: extensor tibiae; Flx: flexor tibiae; Lev: levator trochanteris; Dep: depressor trochanteris; Pro: protractor coxae; Ret: retractor coxae. Ms, Mt: meso- and metathoracic segment, respectively. The sketch indicates a stick insect with front legs in a flexed (inside) or stretched (outside) position, and the arrow above indicating the turning direction, the arrow on the side the recorded thoracic segment. Note that “+ − = ” above the extensor and below the flexor MN pools denotes that there were examples of increased, decreased and unchanged MN activity.

For turns, insects produce leg-specific changes in kinematics that differ from those observed in a regular straight walking pattern (Strauss and Heisenberg, 1990; Jindrich and Full, 1999; Mu and Ritzmann, 2005; Dürr and Ebeling, 2005; Gruhn et al., 2009; Cruse et al., 2009; Gruhn et al., 2011). Front and middle legs on the inside of the turn typically pull the body into the turning direction, while the outside front, middle and hind legs push the body around the curved path, and the inside hind legs often act as a pivot. Given the fact that limb movements are generally controlled by a large number of different muscles which often act synergistically (Santuz et al., 2019; Akay and Murray 2021), the conclusions drawn from kinematic analysis are limited. However, it seems clear that they are the result from an interplay of centrally generated output with local and intersegmental sensory feedback (Ritzmann and Zill 2017; Bidaye et al., 2018; Mantziaris et al., 2020; Akay and Murray, 2021). Understanding the contribution of each of these components, not only requires to know the anatomical connectivity, but also to understand the contribution of the single components in a reduced but still behaving preparation. For this purpose, larger insects such as the stick insect offer an ideal choice to elucidate the neuronal mechanisms for walking pattern generation and sensorimotor integration. For stick insect turning, known neuronal mechanisms underlying the observed motor flexibility include local, body-side specific changes in processing of sensory feedback and body-side dependent changes in motor output that involve influences on CPG activity of the most proximal ThC leg joint (Gruhn et al., 2016; Schmitz et al., 2019). However, it was unclear, whether the observed changes in the motor output during curve walking of the front legs concern only premotor networks controlling the ThC-joint, or also those of the more distal CTr- and FTi-joints, including the involvement of CPG activity. Our results are summarized in Figure 11.

We found that major differences exist in the turning-related motor output for the MN pools that control the movement of all three main leg joints. The metathoracic ThC-joint shows the same pronounced body side-specific differences during inside and outside steps of the front leg that have been described for the mesothorax (Gruhn et al., 2016): the ProCx MNs in both thoracic segments are strongly activated on the inside and only weakly on the outside, whereas the opposite was observed in the RetCx MNs. At the same time the activity on the inside of the turn is rhythmic and phase-coupled to the FL steps, whereas the activity on the outside is tonic and not phase-coupled. Finally, during inside and outside turning, the changes in MN activity are mediated through influences on CPG activity as shown in our experiments with pharmacological activation of the CPG networks.

In contrast, CTr-joint MNs show no turning-related differences in activity. Independent of the turning direction, the LevTr MN activity is strongly increased, whereas DepTr MN activity ceases. This effect is equally mediated through changes in CPG activity, as the pharmacologically activated pilocarpine rhythm shows an increase of the LevTr MN activity during inside and outside turns, similar to the result without pilocarpine, and sometimes the rhythm even appeared locked in levator phase. The strength of this effect on the activity of the CTr-joint MNs appears to weaken from meso- to metathorax.

MNs of the FTi-joint show both, side-specific differences and thorax segment-specific differences in activation, however to a much lesser extent. In contrast to the two more proximal joints, FTi-joint MN activity is highly variable and does not show systematic task-specific changes. Whereas on the outside of the mesothorax, ExtTi MN activity increases compared to FlxTi MN activity, the opposite occurs on the inside. In 50% of the experiments, however, walking sequences with equal activity between contralateral sides were found for both MN pools. In the metathorax, the activity of the ExtTi and FlxTi MN pools increased or decreased in both turning situations, and contralateral sides showed often similar activity in response to FL turning (Figure 11). Overall, we thus present two major findings: 1) the turning-related motor output differs strongly among the three major leg joints and also between the ipsilateral meso- and metathoracic hemisegments; 2) the turning-related effect to the thoracic CPGs decreases from the meso- to the metathorax.

### Motor Control Mechanisms During Turning

Our results highlight the independent control of the different leg joints and thoracic segments in the stick insect (Büschges et al., 1995; Akay et al., 2001; Fischer et al., 2001; Bucher et al., 2003; Borgmann et al., 2012; Büschges and Borgmann, 2013), and show that this relative independence persists during turning, if not even being strengthened. Particularly pronounced changes during turning of the stick insect occur in the leg kinematics at the level of the ThC-joint in *protractor* (ProCx) and *retractor coxae* (RetCx) muscle activation during different walking conditions (Dürr and Ebeling, 2005; Cruse et al., 2009; Rosenbaum et al., 2010; Gruhn et al., 2011). The inside hind leg has been shown to produce on average smaller front to back excursions than the ipsilateral middle leg, and it may act like a pivot, around which the animal turns, when the curve is tight. The outside hind leg moves with a strong front to back excursion, similar to the mesothoracic outside leg (Dürr and Ebeling, 2005; Cruse et al., 2009; Gruhn et al., 2009), which is in line with a variety of other walking insect and crustacean species (e.g., Frantsevitch and Mokrushov, 1980; Strauss and Heisenberg, 1990; Cruse and Saavedra, 1996; Frantsevich and Cruse, 2005; Mu and Ritzmann, 2005).

Under experimental conditions, when a front leg is stepping straight on a treadmill, ipsilateral metathoracic ThC-motor neurons have been reported to become mostly tonically active, and ProCx MNs receive phasic modulatory input in the meso- and to a lesser extent in the metathorax from the stepping front leg (Borgmann et al., 2007). Outside activity is never phase-coupled to the front leg, neither in the meso- nor in the metathoracic ThC-joint. Finally, during inside steps of the front leg, both, meso- and metathoracic ThC-neurons receive phasic signals from the front leg and/or the premotor networks governing FL MNs (Gruhn et al., 2016). Together with our findings, this implies that there is a gradient of descending influence that creates the characteristic motor output from outside to straight, and finally to inside steps. The tonic activity in the metathorax during front leg outside and straight stepping points to the potential importance of local sensory feedback during these two situations, and suggests that the influence from the stepping front leg onto the metathoracic ProCx and RetCx MNs might be weaker than in the mesothorax (Borgmann et al., 2007). When, however, the middle leg is present (but not stepping), rhythmic activation of ProCx and RetCx MN activity through FL stepping can be observed (Borgmann et al., 2009). This will be discussed further below. All in all, however, the similarity in the activity between the two posterior thoracic segments suggests a very similar central control mechanism for this joint.

In the CTr-joint, we observed a lack of the occurrence of turning-direction specific motor patterns. This may be related to the fact that each leg, independent of the direction the animal turns into and even irrespective of any condition the stick insect may encounter, needs to be off the ground during swing and on the ground during stance. The tonic activity of the LevTr MNs and the lack of activity in the DepTr MNs, resembles the situation in the deafferented outside turning ThC-joint MNs, with strong tonic RetCx MN activity and weak to absent ProCx MN activity. Interestingly, in the CTr-joint the swing MNs are tonically active, whereas in the ThC-joint stance MNs are, A strong tonic activation of middle leg levator, and a suppression of its antagonist the depressor has also been reported for the straight stepping single FL preparation (Borgmann et al., 2012). This motor output changed as soon as the middle leg campaniform sensilla (CS) were stimulated. The load stimulus terminated LevTr and initiated DepTr MN activity. This suggests that local load feedback may be crucial for the switch between the antagonists in the CTr-joint irrespective of leg function. Based on the strong effect of load stimuli, and the lack of patterning influence from the front leg, Borgmann et al. (2012) suggested a hierarchy in the strength of inter-leg influence onto the local pattern generating networks which decreases from proximal to distal joints. Our results provide further evidence for this hypothesis, and go beyond, by suggesting that such a gradient may also exist from rostral to caudal between the meso- and metathoracic segments, as we could show that the observed suppression of depressor activity was reduced in the metathoracic CTr-joint. Borgmann et al. (2012) and our results differ from a study by Ludwar et al. (2005a), who reported that front leg stepping activated DepTr MN activity in the deafferented mesothorax which was also phase-coupled to the front leg in six out of nine experiments. Currently, we have no explanation for this discrepancy. The only difference between the approach in Ludwar et al. (2005a) and that in Borgmann et al. (2012) was that in the latter study, the mesothoracic ganglion was not deafferented, while the only difference between Ludwar et al. (2005a) and our study was that walking in our approach happened with reduced sensory input from the front leg due to the use of a slippery surface. Thus, it could be that load information from the front leg plays a different role as soon as local sensory feedback is missing.

The lack of a clear, turning-related motor output of the FTi-joint MNs to the flexor and extensor tibiae muscles further supports the idea of a weaker central drive to the more distal joints and even more so in the more caudal metathoracic segment. Similar to our results, Hellekes et al. (2012) reported a strong, turning-related mesothoracic ExtTi MN output during outside, and strong mesothoracic FlxTi MN output during inside turns of the front legs, when other legs were also still attached. This is likely due to central drive, which is also confirmed by the results of our intracellular recordings where 66% of the ExtTi MNs were more strongly depolarized during outside vs. inside turns. No comparable data exist for the metathoracic MN pools, but it is plausible that intracellular data from the metathoracic FTi-joint MN pools would show more variability in this segment.

### Involvement of Central Pattern Generating Networks in the Expression of Turning-Related Motor Output

The finding that changes in motor output of the deafferented meso- and metathoracic ganglia during FL turning were leg-segment, and partly thorax-segment-specific raised the possibility that not all changes in the meso- and metathorax were mediated through modification of local CPG activity as is the case for the mesothoracic ThC-joint (Gruhn et al., 2016). In arthropods, the muscarinic agonist pilocarpine has long been used to induce fictive locomotion (crayfish: Chrachri and Clarac, 1990; locust: Ryckebusch and Laurent, 1993; hawkmoth: Johnston and Levine, 2002; cockroach: Fuchs et al., 2011) or to elicit rhythmic motoneuronal patterns in individual MN pools (stick insect: Büschges et al., 1995), and demonstrated that each of the three major stick insect leg joints in each leg is controlled by an individual CPG (Büschges et al., 1995). Therefore, we hypothesized that if pilocarpine-elicited CPG activity were altered during front leg curve stepping, it could be concluded that CPGs are involved in the turning related changes in motor output.

The changes in the pilocarpine-induced rhythm of the metathoracic ThC-joint CPG during turning, were body-side specific, similar to the changes during control conditions, and similar to the changes reported for the mesothoracic ProCx- and RetCx MN pools by Gruhn et al. (2016). During inside steps the rhythm was phase-coupled to the front leg step cycle, albeit to a lesser extent than in the mesothorax. On the outside, RetCx MN bursts were lengthened, and not systematically coupled to the FL steps. DepTr MN bursts were always strongly suppressed, and LevTr MN bursts lengthened during stepping sequences independent of the walking direction. In the metathorax, occasional alternation of levator and depressor bursts occurred during walking sequences. The high variability in the MN pools of the FTi-joint during front leg turning under control conditions, even within a single animal, and thus the lack of a good reference, prevented us to study potential changes in the activity of the FTi-joint CPG.

Borgmann et al. (2009) reported that pilocarpine-elicited activity of the ThC-joint MNs was similar in the meso- and metathorax, with strong entrainment of the patterned CPG activity by the stepping of a single front leg on a tread wheel with a phase preference between 300° and 330°. This effect of FL straight stepping onto the metathoracic ipsilateral thoracic ThC-joint CPGs resembles the effect we observed during inside stepping. Thus, during inside stepping the decreasing gradient in activation strength postulated for the meso- and metathoracic ThC-joint by Borgmann et al. (2007) appears to be less prominent than during straight stepping. This may be due to a more prominent role of sensory input based on the FL kinematics during inside turns. However, as mentioned above, Borgmann et al. (2009) also showed that the presence of the middle leg allows the activation of alternating metathoracic ProCx and RetCx MN activity in the absence of pharmacological CPG activation, demonstrating the importance of additional local sensory input for the activation of the CPGs *in vivo*.

For the next distal CTr-joint, a gradient in activation strength during FL turning steps is apparent even during pilocarpine-induced CPG activation. The CTr-joint pilocarpine rhythm is shaped towards the same activity as in regular saline, namely stronger burst in the LevTr MNs. The fact that the typical pilocarpine alternation is still occurring in the metathoracic CTr-joint CPG during FL curve stepping demonstrates that any descending input preventing the alternating activity in the mesothorax is weaker in the metathorax, and supports our hypothesis according to which the descending influence that coincides with the FL curve stepping is weaker in the meta- compared to the mesothorax. The only data that exist so far on intersegmental influences in the pilocarpine activated CTr-joint MNs in the stick insect are from Mantziaris et al. (2017), Daun et al. (2019), and Mantziaris et al. (2020), who studied the central coupling between the three thoracic CTr CPGs without descending input from the head ganglia, and after complete deafferentation of all ganglia. Mantziaris et al. did observe weak coupling between ipsilateral CPGs of the same joint and proposed that central pathways between them exist that exert a coordinating influence. Furthermore, Daun et al. (2019) found that the central coupling between the prothoracic and the meso- or metathoracic ganglia was generally weaker than coupling between the two more posterior ganglia. This suggests that turning-related changes that are seen in the meso- and metathoracic CTr-joint MN activity are likely not to come from the activity of the FL CPGs, otherwise we would expect a patterned influence coupled to the FL steps. The neural control that alters CPG activity in the CTr-joint is thus more likely to derive from neural networks or inputs arising anterior to the thoracic ganglia. This, however, stresses how crucial local sensory feedback is for the generation of a functional motor pattern. Interestingly, a study by Knebel et al. (2017) in the locust reported a strong coupling between the pro- and mesothoracic ganglia after pharmacological activation, suggesting a less flexible control of leg movements in this species.

### Interplay of Central and Sensory Inputs for the Motor Output During Turning

The motor output in the deafferented meso- and metathoracic ganglia does not resemble a functional motor output observed during walking of an intact animal. Translated into actual movement, the inside middle and hind leg motor output would produce a front-to-back movement of the lifted leg, together with occasional flexion movements around the FTi-joint. The outside legs would be permanently retracted, lifted, and most likely also stretched. It appears that this output only represents a necessary default activation, on which each joint depends to perform the kinematic changes observed during turning *in vivo*. The pronounced differences in the motor output to the ThC-joint possibly relate to the very strong alterations in the forward and backward movement of the inside and outside legs leading to changes in anterior and posterior extreme positions (AEP, PEP) (Dürr and Ebeling, 2005; Gruhn et al., 2009). The lack of a body side-specific activity of the CTr-joint MN pools points to the importance of this joint to determine stance and swing, independent of any direction, while the observed activation patterns in the FTi-joint fits to the role of the flexor as a stance and the extensor as a swing phase muscle, independent of the body side. They also fit to the role of both muscles to serve body height control through co-activation, but also reflect the relatively large kinematic variability in this joint during turning (Dürr and Ebeling, 2005; Gruhn et al., 2009).

This stresses the importance of an integration of CPG activity with sensory input in order to shape the functional motor output *in vivo*. According to our results, the activity of the different joint MN pools suggests that the impact of central descending influences on the motor output weakens while the dependence of motor output on local sensory feedback increases from rostral to caudal and from proximal to distal. This importance of local sensory feedback on timing and magnitude of the motor output is known from the stick insect and has been studied in great detail (Hess and Büschges, 1997; Hess and Büschges, 1999; Akay et al., 2001; Bucher et al., 2003; Akay et al., 2004; Büschges and Gruhn, 2008; Gebehart et al., 2021; Gebehart and Büschges 2021). Interaction of central and peripheral influences for the generation of functional motor outputs are known from crustaceans and stick insects (Sillar et al., 1987; Bucher et al., 2003; Borgmann et al., 2009; Borgmann et al., 2012; Gebehart and Büschges 2021; Gebehart et al., 2021), and may even affect the timing and strength of MN activity in the adjacent thoracic segment (Sillar et al., 1987). At present, however, due to the experimental restrictions of our study, we cannot be conclusive about the interactions between central and peripheral inputs and their weighing at the level of each joint during turning.

In the meso- and the metathorax, the ThC-joint MNs of the inside leg, even when completely deafferented, show a rhythmic, alternating output that is in phase with the front leg step cycle. For this joint and this turning direction, the dependence on local feedback appears to be necessary to uncouple the two neighboring legs from one another. The outside ThC-joint MNs are often not switching from RetCx to ProCx MN activity, but the activity appears to be locked in RetCx phase of activity. Here, a strong dependence on load feedback has been postulated for the mesothorax, based on the alteration of the processing of load feedback in this segment during turning (Gruhn et al., 2016) and is likely to be present in the metathorax as well.

The absence of alternating activity between LevTr and DepTR MN pools of the CTr-joint resembles the outside activity of the ThC-joint MN pools, and implies a similar dependence of CPG activity on local sensory input during turning-related movements. This dependence on local sensory input in the CTr-joint has been known to arise from load signals from the campaniform sensilla (CS) (Akay et al., 2007; Rosenbaum et al., 2010; Borgmann et al., 2012; Zill et al., 2015; Gebehart et al., 2021), as well as feedback from the femoral chordotonal organ (fCO), which monitors position- and movement of the tibia (Hess and Büschges, 1999; Bucher et al., 2003; Gebehart et al., 2021). It is possible that the processing of this input, which has been shown to have access to the CTr-joint CPG, is modified during turning, similar to the load feedback in the mesothoracic ThC-joint MNs (Gruhn et al., 2016). The lack of a difference in the CTr-joint MN activity of the two body sides may be due to the difference in the functional role of the joint, as the leg has always to be on the ground during stance independent of its role as an outside or inside leg.

The apparent failure of the deafferented preparation to produce a consistent turning-related output in FTi-joint MNs, which is especially evident in the metathorax, suggests a weakening of turning-related intersegmental and central influences towards the metathorax for this joint as well. From the extracellular recordings, it appears that activity modification during turning is much weaker and less consistent in the metathoracic FTi-joint MNs, probably because the central drive has a weaker effect. The importance of sensory feedback in the FTi-joint control system has also long been known (Graham and Bässler, 1981; Hofmann et al., 1985; Bässler, 1993; Akay et al., 2001; Schmitz et al., 2015; Zill et al., 2015; Zill et al., 2017). Schmitz et al. (2015) observed in a different experimental setup, that 19% of middle leg steps into a hole without ground contact failed to elicit flexor tibiae EMG activity. They attributed the observed effect to the failure of local load feedback to drive FlxTi MN, which are known to be activated above firing threshold by tarsal touchdown (Gruhn et al., 2006; Berendes et al., 2013; Schmitz et al., 2015). We also know that signaling of movement feedback from the fCO in the mesothoracic ganglion is locally processed during turning to promote active flexion in the inside, but not in the outside leg (Hellekes et al., 2012). Furthermore, Gebehart and Büschges (2021) have recently shown that temporal differences in the reporting of load and movement feedback are a crucial factor for the integration of this sensory information in the pre-motor interneurons. Therefore, absence of sensory feedback in the deafferented preparation we used may have contributed to the observed variability in motor output to this joint. The presumed increase in dependence on local sensory input towards the metathorax has been mentioned above and seems to be valid for all three joints. Given the proximity of the metathoracic legs to the center of mass of the stick insect body (Cruse, 1976; Dallmann et al., 2016), it is conceivable that the necessity of load feedback for the patterning may be even more pronounced for the metathoracic than the mesothoracic motor circuits. So far, however, we have no information about the influence of load feedback in the metathoracic walking legs and future studies should focus on such sensory influences on the metathoracic motor activity.

In summary, we have presented evidence showing that the motor output to the three main leg joints of the meso- and the metathoracic legs of the stick insect, i.e., the ThC, the CTr-, and the FTi-joints, during turning is not only joint-specific, but also differs depending on the thoracic segment. We show that changes in the activity during turning are most likely mediated by influences on local CPG activities, and that the respective influences on segmental CPGs weaken caudally towards the metathorax. We conclude from our results that the turning-related motor output strongly depends on local or inter-leg sensory feedback, in order to be shaped into the functional inside or outside stepping pattern observed in the intact behaving stick insect. Future experiments will have to address the sources of this descending influence that is causal for the observed activity changes during turning, the mechanisms behind the decreasing influence of the central drive, and the change in local sensory processing observed in the mesothoracic ThC- and FTi- joints.

## Data Availability

The raw data supporting the conclusion of this article will be made available by the authors, without undue reservation.

## Supplementary Material

Supplementary Figure S1 Two examples each of the metathoracic *extensor* **(A–C)** and *flexor tibiae* **(D–F)** MN output in the deafferented ganglion during front leg (FL) inside and outside stepping. **(A)** flexor EMG of the front leg (top trace and third trace), together with extracellular recordings of the ipsilateral metathoracic extensor nerve (nl3) during inside (i,iii) and outside (ii,iv) stepping sequences; **(Bi,ii)** Phase histograms of extensor nerve activity from the walking sequences in **(Ai,ii)** and **(Aiii,iv)**, comparing inside (I) and outside (O) activity in the same animal with respect to FL step cycle. **(C)** Polar plots with spike event maxima (grey vectors) and their mean (red) for the extensor with respect to FL step cycle during inside (top, I) and outside (bottom, O) stepping; **(D)** flexor EMG recording of the FL (top trace and third trace), together with extracellular recordings of the ipsilateral metathoracic flexor nerve (ncr) during inside (i,iii) and outside (ii,iv) stepping sequences; **(Ei,ii)**. Phase histograms of flexor nerve activity from experiments in **(Di,ii)** and **(Diii,iv)**, comparing inside (I) and outside (O) activity in the same animal with respect to FL step cycle. **(F)** Polar plots with spike event maxima (grey vectors) and their mean (red) for the flexor with respect FL step cycle during inside (top, I) and outside (bottom, O) stepping; N = number animals; Ń = number of analyzed hemiganglia; *n* = number of analyzed steps. Pr, Mt: Pro- and Metathoracic location of the recording, respectively; Flex/FlxTi, Ext/ExtTi: *flexor tibiae* and *extensor tibiae* activity, respectively. The sketch indicates a stick insect with front legs in a flexed (inside) or stretched (outside) position, and the arrow above indicating the turning direction, the arrow on the side the recorded thoracic segment.

## References

[B1] Akay T. Murray A. J. (2021). Relative Contribution of Proprioceptive and Vestibular Sensory Systems to Locomotion: Opportunities for Discovery in the Age of Molecular Science. Int. J. Mol. Sci. 22 (3), 1467–1485. 10.3390/ijms22031467 33540567 PMC7867206

[B2] Akay T. Bässler U. Gerharz P. Büschges A. (2001). The Role of Sensory Signals from the Insect Coxa-Trochanteral Joint in Controlling Motor Activity of the Femur-Tibia Joint. J. Neurophysiology 85, 594–604. 10.1152/jn.2001.85.2.594 11160496

[B3] Akay T. Haehn S. Schmitz J. Büschges A. (2004). Signals from Load Sensors Underlie Interjoint Coordination during Stepping Movements of the Stick Insect Leg. J. Neurophysiol. 92, 42–51. 10.1152/jn.01271.2003 14999042

[B4] Akay T. Ludwar B. C. Göritz M. L. Schmitz J. Büschges A. (2007). Segment Specificity of Load Signal Processing Depends on Walking Direction in the Stick Insect Leg Muscle Control System. J. Neurosci. 27, 3285–3294. 10.1523/jneurosci.5202-06.2007 17376989 PMC6672458

[B5] Akay T. (2020). Sensory Feedback Control of Locomotor Pattern Generation in Cats and Mice. Neuroscience 450, 161–167. 10.1016/j.neuroscience.2020.05.008 32422335

[B6] Ampatzis K. Song J. Ausborn J. El Manira A. (2014). Separate Microcircuit Modules of Distinct V2a Interneurons and Motoneurons Control the Speed of Locomotion. Neuron 83 (4), 934–943. 10.1016/j.neuron.2014.07.018 25123308

[B7] Ausborn J. Mahmood R. El Manira A. (2012). Decoding the Rules of Recruitment of Excitatory Interneurons in the Adult Zebrafish Locomotor Network. Proc. Natl. Acad. Sci. U. S. A. 109 (52), E3631–E3639. 10.1073/pnas.1216256110 23236181 PMC3535644

[B8] Bässler U. Büschges A. (1998). Pattern Generation for Stick Insect Walking Movements-Multisensory Control of a Locomotor Program. Brain Res. Rev. 27, 65–88. 10.1016/s0165-0173(98)00006-x 9639677

[B9] Bässler U. Storrer J. (1980). The Neural Basis of the Femur-Tibia-Control-System in the Stick Insect Carausius Morosus. Biol. Cybern. 38, 107–114. 10.1007/bf00356037

[B10] Bässler U. Wegner U. T. A. (1983). Motor Output of the Denervated Thoracic Ventral Nerve Cord in the Stick Insect *Carausius Morosus* . J. Exp. Biol. 105, 127–145. 10.1242/jeb.105.1.127

[B11] Bässler U. (1977). Sensory Control of Leg Movement in the Stick Insect *Carausius Morosus* . Biol. Cybern. 25, 61–72. 10.1007/bf00337264 836915

[B12] Bässler U. (1993). The Femur-Tibia Control System of Stick Insects - A Model System for the Study of the Neural Basis of Joint Control. Brain Res. Rev. 18, 207–226. 10.1016/0165-0173(93)90002-h 8339107

[B13] Bender J. A. Simpson E. M. Tietz B. R. Daltorio K. A. Quinn R. D. Ritzmann R. E. (2011). Kinematic and Behavioral Evidence for a Distinction between Trotting and Ambling Gaits in the cockroachBlaberus Discoidalis. J. Exp. Biol. 214 (12), 2057–2064. 10.1242/jeb.056481 21613522 PMC3102013

[B14] Berendes V. Dübbert M. Bockemühl T. Schmitz J. Büschges A. Gruhn M. (2013). A Laser-Supported Lowerable Surface Setup to Study the Role of Ground Contact during Stepping. J. Neurosci. Methods 215, 224–233. 10.1016/j.jneumeth.2013.03.024 23562598

[B15] Berens P. (2009). CircStat: A MATLAB Toolbox for Circular Statistics. J. Stat. Softw. 31, 1–21. 10.18637/jss.v031.i10

[B16] Bidaye S. S. Bockemühl T. Büschges A. (2018). Six-legged Walking in Insects: How CPGs, Peripheral Feedback, and Descending Signals Generate Coordinated and Adaptive Motor Rhythms. J. Neurophysiol. 119, 459–475. 10.1152/jn.00658.2017 29070634

[B17] Bidaye S. S. Laturney M. Chang A. K. Liu Y. Bockemühl T. Büschges A. (2020). Two Brain Pathways Initiate Distinct Forward Walking Programs in Drosophila. Neuron 108 (3), 469–485. 10.1016/j.neuron.2020.07.032 32822613 PMC9435592

[B18] Borgmann A. Scharstein H. Büschges A. (2007). Intersegmental Coordination: Influence of a Single Walking Leg on the Neighboring Segments in the Stick Insect Walking System. J. Neurophysiol. 98, 1685–1696. 10.1152/jn.00291.2007 17596420

[B19] Borgmann A. Hooper S. L. Büschges A. (2009). Sensory Feedback Induced by Front-Leg Stepping Entrains the Activity of Central Pattern Generators in Caudal Segments of the Stick Insect Walking System. J. Neurosci. 29, 2972–2983. 10.1523/jneurosci.3155-08.2009 19261892 PMC6666218

[B20] Borgmann A. Toth T. I. Gruhn M. Daun-Gruhn S. Büschges A. (2012). Dominance of Local Sensory Signals over Inter-segmental Effects in a Motor System: Experiments. Biol. Cybern. 105, 399–411. 10.1007/s00422-012-0473-y 22290138

[B93] Brunner von Wattenwyl K. (1907). Die Insektenfamilie der Phasmiden. Leipzig: Wilhelm Engelmann, Vol. 2.

[B27] Bucher D. Akay T. Dicaprio R. A. Büschges A. (2003). Interjoint Coordination in the Stick Insect Leg-Control System: the Role of Positional Signaling. J. Neurophysiol. 89, 1245–1255. 10.1152/jn.00637.2002 12626610

[B21] Büschges A. Borgmann A. (2013). Network Modularity: Back to the Future in Motor Control. Curr. Biol. 23, R936–R938. 10.1016/j.cub.2013.09.021 24156817

[B22] Büschges A. Schmidt J. (2015). Neuronal Control of Walking: Studies on Insects. e-Neuroforum 6, 105–112. 10.1007/s13295-015-0017-8

[B23] Büschges A. Schmitz J. (1991). Nonspiking Pathways Antagonize the Resistance Reflex in the Thoraco-Coxal Joint of Stick Insects. J. Neurobiol. 22 (3), 224–237. 10.1002/neu.480220303 1890415

[B24] Büschges A. Schmitz J. Bässler U. (1995). Rhythmic Patterns in the Thoracic Nerve Cord of the Stick Insect Induced by Pilocarpine. J. Exp. Biol. 198, 435–456. 10.1242/jeb.198.2.435 9318078

[B25] Büschges A. Akay T. Gabriel J. P. Schmidt J. (2008). Organizing Network Action for Locomotion: Insights from Studying Insect Walking. Brain Res. Rev. 57, 162–171. 10.1016/j.brainresrev.2007.06.028 17888515

[B26] Büschges A. (1998). Inhibitory Synaptic Drive Patterns Motoneuronal Activity in Rhythmic Preparations of Isolated Thoracic Ganglia in the Stick Insect. Brain Res. 783, 262–271. 10.1016/s0006-8993(97)01370-x 9507159

[B28] Büschges A. Gruhn M. (2008). Mechanosensory Feedback in Walking: From Joint Control to Locomotor Patterns. Adv. Insect Physiol. 34, 193–230. 10.1016/S0065-2806(07)34004-6

[B29] Büschges A. Ludwar B. C. Bucher D. Schmidt J. Dicaprio R. A. (2004). Synaptic Drive Contributing to Rhythmic Activation of Motoneurons in the Deafferented Stick Insect Walking System. Eur. J. Neurosci. 19, 1856–1862. 10.1111/j.1460-9568.2004.03312.x 15078559

[B31] Chrachri A. Clarac F. (1990). Fictive Locomotion in the Fourth Thoracic Ganglion of the Crayfish, *Procambarus clarkii* . J. Neurosci. 10, 707–719. 10.1523/jneurosci.10-03-00707.1990 2319299 PMC6570116

[B32] Cruse H. Saavedra M. (1996). Curve Walking in Crayfish. J. Exp. Biol. 199 (7), 1477–1482. 10.1242/jeb.199.7.1477 9319377

[B33] Cruse H. Ehmanns I. Stübner S. Schmitz J. (2009). Tight Turns in Stick Insects. J. Comp. Physiol. A 195, 299–309. 10.1007/s00359-008-0406-3 19137316

[B34] Cruse H. (1976). The Function of the Legs in the Free Walking Stick insect,Carausius Morosus. J. Comp. Physiol. 112, 235–262. 10.1007/bf00606541

[B35] Dallmann C. J. Dürr V. Schmitz J. (2016). Joint Torques in a Freely Walking Insect Reveal Distinct Functions of Leg Joints in Propulsion and Posture Control. Proc. Biol. Sci. 283, 20151708. 10.1098/rspb.2015.1708 26791608 PMC4795010

[B36] Daun S. Mantziaris C. Tóth T. Büschges A. Rosjat N. (2019). Unravelling Intra- and Intersegmental Neuronal Connectivity between Central Pattern Generating Networks in a Multi-Legged Locomotor System. PLOS ONE 14 (8), e0220767. 10.1371/journal.pone.0220767 31386699 PMC6684069

[B37] Dean J. (1989). Leg Coordination in the Stick Insect Carausius Morosus: Effects of Cutting Thoracic Connectives. J. Exp. Biol. 145, 103–131. 10.1242/jeb.145.1.103

[B38] Deangelis B. D. Zavatone-Veth J. A. Clark D. A. (2019). The Manifold Structure of Limb Coordination in Walking Drosophila. eLife 8, 46409ff. 10.7554/eLife.46409 PMC6598772 31250807

[B39] Dürr V. Ebeling W. (2005). The Behavioural Transition from Straight to Curve Walking: Kinetics of Leg Movement Parameters and the Initiation of Turning. J. Exp. Biol. 208, 2237–2252. 10.1242/jeb.01637 15939767

[B40] Dürr V. Theunissen L. M. Dallmann C. J. Hoinville T. Schmitz J. (2018). Motor Flexibility in Insects: Adaptive Coordination of Limbs in Locomotion and Near-Range Exploration. Behav. Ecol. Sociobiol. 72, 15. 10.1007/s00265-017-2412-3

[B41] Field E. C. Stein P. S. G. (1997a). Spinal Cord Coordination of Hindlimb Movements in the Turtle: Interlimb Temporal Relationships during Bilateral Scratching and Swimming. J. Neurophysiology 78, 1404–1413. 10.1152/jn.1997.78.3.1404 9310431

[B42] Field E. C. Stein P. S. G. (1997b). Spinal Cord Coordination of Hindlimb Movements in the Turtle: Intralimb Temporal Relationships during Scratching and Swimming. J. Neurophysiology 78, 1394–1403. 10.1152/jn.1997.78.3.1394 9310430

[B43] Fischer H. Schmidt J. Haas R. Büschges A. (2001). Pattern Generation for Walking and Searching Movements of a Stick Insect Leg. I. Coordination of Motor Activity. J. Neurophysiol. 85, 341–353. 10.1152/jn.2001.85.1.341 11152734

[B44] Frantsevich L. I. Cruse H. (2005). Leg Coordination during Turning on an Extremely Narrow Substrate in a Bug, *Mesocerus Marginatus* (Heteroptera, Coreidae). J. Insect Physiol. 51, 1092–1104. 10.1016/j.jinsphys.2005.05.008 16162355

[B45] Frantsevitch L. I. Mokrushov P. A. (1980). Turning and Righting in *Geotrupes* (Coleoptera, Scarabaeidae). J. Comp. Physiol. A 136 (4), 279–289.

[B46] Fuchs E. Holmes P. Kiemel T. Ayali A. (2011). Intersegmental Coordination of Cockroach Locomotion: Adaptive Control of Centrally Coupled Pattern Generator Circuits. Front. Neural Circuits 4, 125. 10.3389/fncir.2010.00125 21369365 PMC3043608

[B47] Gebehart C. Büschges A. (2021). Temporal Differences between Load and Movement Signal Integration in the Sensorimotor Network of an Insect Leg. J. Neurophysiol. 126 (6), 1875–1890. 10.1152/jn.00399.2021 34705575

[B48] Gebehart C. Schmidt J. Büschges A. (2021). Distributed Processing of Load and Movement Feedback in the Premotor Network Controlling an Insect Leg Joint. J. Neurophysiol. 125 (5), 1800–1813. 10.1152/jn.00090.2021 33788591

[B49] Godden D. H. (1972). The Motor Innervation of the Leg Musculature and Motor Output during Thanatosis in the Stick insectCarausius Morosus Br. J. Comp. Physiol. 80, 201–225. 10.1007/bf00696491

[B50] Goldammer J. Büschges A. Schmidt J. (2012). Motoneurons, DUM Cells, and Sensory Neurons in an Insect Thoracic Ganglion: a Tracing Study in the Stick Insect Carausius Morosus. J. Comp. Neurol. 520, 230–257. 10.1002/cne.22676 21618233

[B51] Graham D. Bässler U. (1981). Effects of Afference Sign Reversal on Motor Activity in Walking Stick Insects (*Carausius Morosus*). J. Exp. Biol. 91, 179–193. 10.1242/jeb.91.1.179

[B52] Graham D. Wendler G. (1981). The Reflex Behaviour and Innervation of the Tergo-Coxal Retractor Muscles of the Stick insectCarausius Morosus. J. Comp. Physiol. 143, 81–91. 10.1007/bf00606071

[B53] Graham D. (1985). “Pattern and Control of Walking in Insects,” in Advances in Insect Physiology (London: Academic Press), 31–140. 10.1016/s0065-2806(08)60039-9

[B54] Grillner S. (2003). The Motor Infrastructure: from Ion Channels to Neuronal Networks. Nat. Rev. Neurosci. 4 (7), 573–586. 10.1038/nrn1137 12838332

[B55] Gruhn M. Hoffmann O. Dübbert M. Scharstein H. Büschges A. (2006). Tethered Stick Insect Walking: a Modified Slippery Surface Setup with Optomotor Stimulation and Electrical Monitoring of Tarsal Contact. J. Neurosci. Methods 158, 195–206. 10.1016/j.jneumeth.2006.05.029 16824615

[B56] Gruhn M. Zehl L. Büschges A. (2009). Straight Walking and Turning on a Slippery Surface. J. Exp. Biol. 212, 194–209. 10.1242/jeb.018317 19112138

[B57] Gruhn M. Rosenbaum P. Bollhagen H. P. Büschges A. (2011). Studying the Neural Basis of Adaptive Locomotor Behavior in Insects. J. Vis. Exp. 50, e2629. 10.3791/2629 PMC3169265 21525839

[B58] Gruhn M. Rosenbaum P. Bockemühl T. Büschges A. (2016). Body Side-specific Control of Motor Activity during Turning in a Walking Animal. eLife 5, e13799. 10.7554/eLife.13799 27130731 PMC4894755

[B59] Guo P. Ritzmann R. E. (2013). Neural Activity in the Central Complex of the Cockroach Brain Is Linked to Turning Behaviors. J. Exp. Biol. 216, 992–1002. 10.1242/jeb.080473 23197098

[B60] Hellekes K. Blincow E. Hoffmann J. Büschges A. (2012). Control of Reflex Reversal in Stick Insect Walking: Effects of Intersegmental Signals, Changes in Direction, and Optomotor-Induced Turning. J. Neurophysiol. 107, 239–249. 10.1152/jn.00718.2011 21994271

[B61] Hess D. Büschges A. (1997). Sensorimotor Pathways Involved in Interjoint Reflex Action of an Insect Leg. J. Neurobiol. 33, 891–913. 10.1002/(sici)1097-4695(199712)33:7<891::aid-neu3>3.0.co;2-3 9407012

[B62] Hess D. Büschges A. (1999). Role of Proprioceptive Signals from an Insect Femur-Tibia Joint in Patterning Motoneuronal Activity of an Adjacent Leg Joint. J. Neurophysiol. 81, 1856–1865. 10.1152/jn.1999.81.4.1856 10200220

[B63] Hofmann T. Koch U. T. Bässler U. (1985). Physiology of the Femoral Chordotonal Organ of the Stick Insect *Cuniculina Impigra* . J. Exp. Biol. 114, 207. 10.1242/jeb.114.1.207

[B64] Jindrich D. L. Full R. J. (1999). Many-legged Maneuverability: Dynamics of Turning in Hexapods. J. Exp. Biol. 202 (Pt 12), 1603–1623. 10.1242/jeb.202.12.1603 10333507

[B65] Johnston R. Levine R. (2002). Thoracic Leg Motoneurons in the Isolated CNS of Adult Manduca Produce Patterned Activity in Response to Pilocarpine, Which Is Distinct from that Produced in Larvae. Invertebr. Neurosci. 4, 175–192. 10.1007/s10158-002-0019-4 12488968

[B66] Knebel D. Ayali A. Pflüger H.-J. Rillich J. (2017). Rigidity and Flexibility: The Central Basis of Inter-leg Coordination in the Locust. Front. Neural Circuits 10, 112. 10.3389/fncir.2016.00112 28123358 PMC5225121

[B67] Ludwar B. C. Göritz M. L. Schmidt J. (2005a). Intersegmental Coordination of Walking Movements in Stick Insects. J. Neurophysiol. 93, 1255–1265. 10.1152/jn.00727.2004 15525808

[B68] Ludwar B. C. Westmark S. Büschges A. Schmidt J. (2005b). Modulation of Membrane Potential in Mesothoracic Moto- and Interneurons during Stick Insect Front-Leg Walking. J. Neurophysiol. 94, 2772–2784. 10.1152/jn.00493.2005 16000520

[B69] Mantziaris C. Bockemühl T. Holmes P. Borgmann A. Daun S. Büschges A. (2017). Intra- and Intersegmental Influences Among Central Pattern Generating Networks in the Walking System of the Stick Insect. J. Neurophysiol. 118, 2296–2310. 10.1152/jn.00321.2017 28724783 PMC5629271

[B70] Mantziaris C. Bockemühl T. Büschges A. (2020). Central Pattern Generating Networks in Insect Locomotion. Dev. Neurobiol. 80 (1-2), 16–30. 10.1002/dneu.22738 32128970

[B71] Marquart F. (1940). Beiträge zur Anatomie der Muskulatur und der peripheren Nerven von *Carausius* (*Dixipus) morosus* . Zool. Jahrbücher Abt. Anat. Ontol. Tiere 66, 63–128.

[B72] Martin J. P. Guo P. Mu L. Harley C. M. Ritzmann R. E. (2015). Central-complex Control of Movement in the Freely Walking Cockroach. Curr. Biol. 25, 2795–2803. 10.1016/j.cub.2015.09.044 26592340

[B73] Mu L. Ritzmann R. E. (2005). Kinematics and Motor Activity during Tethered Walking and Turning in the Cockroach, *Blaberus Discoidalis* . J. Comp. Physiol. A 191, 1037–1054. 10.1007/s00359-005-0029-x 16258746

[B74] Mu L. Ritzmann R. E. (2008). Interaction between Descending Input and Thoracic Reflexes for Joint Coordination in Cockroach: I. Descending Influence on Thoracic Sensory Reflexes. J. Comp. Physiol. A 194, 283–298. 10.1007/s00359-007-0307-x 18094976

[B75] Pearson K. G. Stein R. B. Malhotra S. K. (1970). Properties of Action Potentials from Insect Motor Nerve Fibres. J. Exp. Biol. 53, 299–316. 10.1242/jeb.53.2.299 5481663

[B76] Pfeffer S. E. Wahl V. L. Wittlinger M. Wolf H. (2019). High-speed Locomotion in the Saharan Silver Ant, Cataglyphis Bombycina. J. Exp. Biol. 222 (29), jeb198705. 10.1242/jeb.198705 31619540

[B77] Ridgel A. L. Alexander B. E. Ritzmann R. E. (2007). Descending Control of Turning Behavior in the Cockroach, Blaberus Discoidalis. J. Comp. Physiol. A 193, 385–402. 10.1007/s00359-006-0193-7 17123086

[B78] Ritzmann R. E. Zill S. N. (2017). “Control of Locomotion in Hexapods,” in The Oxford Handbook of Invertebrate Neurobiology. Editor Byrne J. E. (Oxford University Press. Oxford Handbooks Online). 10.1093/oxfordhb/9780190456757.013.20

[B79] Rosenbaum P. Wosnitza A. Büschges A. Gruhn M. (2010). Activity Patterns and Timing of Muscle Activity in the Forward Walking and Backward Walking Stick InsectCarausius Morosus. J. Neurophysiol. 104, 1681–1695. 10.1152/jn.00362.2010 20668273

[B80] Rosenbaum P. Schmitz J. Schmidt J. Büschges A. (2015). Task-dependent Modification of Leg Motor Neuron Synaptic Input Underlying Changes in Walking Direction and Walking Speed. J. Neurophysiol. 114, 1090–1101. 10.1152/jn.00006.2015 26063769 PMC4725107

[B81] Ryckebusch S. Laurent G. (1993). Rhythmic Patterns Evoked in Locust Leg Motor Neurons by the Muscarinic Agonist Pilocarpine. J. Neurophysiol. 69 (5), 1583–1595. 10.1152/jn.1993.69.5.1583 8389831

[B82] Santuz A. Akay T. Mayer W. P. Wells T. L. Schroll A. Arampatzis A. (2019). Modular Organization of Murine Locomotor Pattern in the Presence and Absence of Sensory Feedback from Muscle Spindles. J. Physiol. 597 (12), 3147–3165. 10.1113/jp277515 30916787

[B83] Schmitz J. Büschges A. Delcomyn F. (1988). An improved electrode design for en passant recording from small nerves. Comp. Biochem. Physiol. Part A Physiol. 91, 769–772. 10.1016/0300-9629(88)90963-2 2907444

[B84] Schmitz J. Gruhn M. Büschges A. (2015). The Role of Leg Touchdown for the Control of Locomotor Activity in the Walking Stick Insect. J. Neurophysiol. 113, 2309–2320. 10.1152/jn.00956.2014 25652931 PMC4416576

[B85] Schmitz J. Gruhn M. Büschges A. (2019). Body Side-specific Changes in Sensorimotor Processing of Movement Feedback in a Walking Insect. J. Neurophysiol. 122 (5), 2173–2186. 10.1152/jn.00436.2019 31553676 PMC6879953

[B86] Schmitz J. (1986). The Depressor Trochanteris Motoneurones and Their Role in the Coxo-Trochanteral Feedback Loop in the Stick Insect *Carausius Morosus* . Biol. Cybern. 55, 25–34. 10.1007/bf00363975

[B87] Sillar K. T. Clarac F. Bush B. M. (1987). Intersegmental Coordination of Central Neural Oscillators for Rhythmic Movements of the Walking Legs in Crayfish, *Pacifastacus Leniusculus* . J. Exp. Biol. 131, 245–264. 10.1242/jeb.131.1.245

[B88] Stein P. S. G. (2018). Central Pattern Generators in the Turtle Spinal Cord: Selection Among the Forms of Motor Behaviors. J. Neurophysiol. 119 (2), 422–440. 10.1152/jn.00602.2017 29070633 PMC5867383

[B94] Strauss R. Heisenberg M. (1990). Coordination of Legs During Straight Walking and Turning in Drosophila Melanogaster. Journal of Comparative Physiology. A, Sensory, Neural, and Behavioral Physiology 167. 10.1007/BF00192575 2121965

[B89] Weidler D. J. Diecke F. P. J. (1969). The Role of Cations in Conduction in the Central Nervous System of the Herbivorous Insect Carausius Morosus. Z. Vergl. Physiol. 64, 372–399. 10.1007/bf00340433

[B90] Westmark S. Oliveira E. E. Schmidt J. (2009). Pharmacological Analysis of Tonic Activity in Motoneurons during Stick Insect Walking. J. Neurophysiol. 102, 1049–1061. 10.1152/jn.91360.2008 19515945

[B91] Zill S. N. Chaudhry S. Büschges A. Schmitz J. (2015). Force Feedback Reinforces Muscle Synergies in Insect Legs. Arthropod Struct. Dev. 44, 541–553. 10.1016/j.asd.2015.07.001 26193626

[B92] Zill S. N. Neff D. Chaudhry S. Exter A. Schmitz J. Büschges A. (2017). Effects of Force Detecting Sense Organs on Muscle Synergies Are Correlated with Their Response Properties. Arthropod Struct. Dev. 46, 564–578. 10.1016/j.asd.2017.05.004 28552666 PMC5817982

