# Supplementary figures and images for: Thorax-Segment- and Leg-Segment-Specific Motor Control for Adaptive Behavior
Source: Front Physiol. 2022 May 4;13:883858. doi: 10.3389/fphys.2022.883858 (PMC9114818; doi:10.3389/fphys.2022.883858)

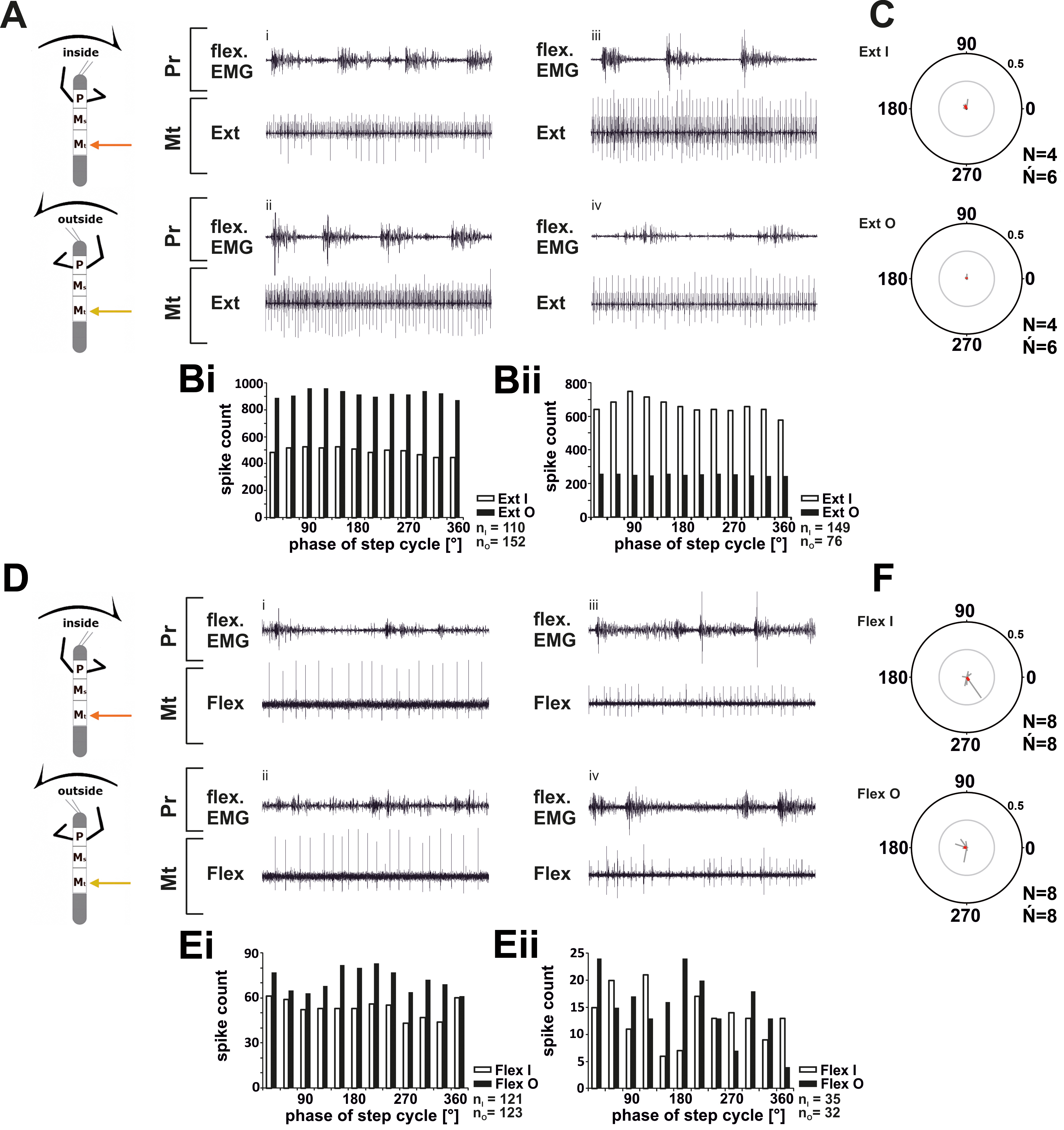

Supplement: Supplementary file 1 [file Image1.JPEG]
